# Supplementary material for: Extent, impact, and mitigation of batch effects in tumor biomarker studies using tissue microarrays
Source: eLife. 2021 Dec 23;10:e71265. doi: 10.7554/eLife.71265 (PMC8849344; doi:10.7554/eLife.71265)
Supplement: Supplementary file 1. — (a) Interaction terms to test for multiplicative effect modification, that is whether batch effects more strongly affect tumors with more extreme clinical/pathological characteristics. The table shows point estimates (differences in biomarker levels), 95% confidence interval bounds, p-values, and false-discovery rates (FDR, in ascending order) for interaction terms between the within-batch normalized biomarker level and the potential effect modifier in linear models that have absolute biomarker levels in standard deviation units per biomarker as the outcome and also include main effects for the biomarker and the effect modifier (terms not shown). (b) ICC for between-batch variance for uncorrected biomarker levels (“1 Raw”) and biomarker levels after applying different correction methods. (c) Results from plasmode simulation according to type of induced batch effect, using the data correlation structure “moderate confounding.” For three fixed (“true”) hazard ratios for the biomarker–outcome association (1/3, 1, and 3), the observed hazard ratios after batch correction (with 95% confidence intervals) are shown. (d) Results from plasmode simulation according to data correlation structure, using the batch effect “mean and variance.” For three fixed (“true”) hazard ratios for the biomarker–outcome association (1/3, 1, and 3), the observed hazard ratios after batch correction (with 95% confidence intervals) are shown. (e) Gleason grade—biomarker associations according to batch effect correction method. Point estimates from unadjusted linear regression models for biomarker values with Gleason score categories per 1 “grade group” increase as the predictor are shown (with 95% confidence intervals). For loge-transformed markers like Ki-67, estimates are differences on the loge scale. (f) Biomarker levels and lethal disease according to batch effect correction method. Hazard ratios (with 95% confidence intervals) per one standard deviation increase in the biomarker (linear) fr [file elife-71265-supp1.docx]

**Supplementary File 1a.** Interaction terms to test for multiplicative effect modification, i.e. whether batch effects more strongly affect tumors with more extreme clinical/pathological characteristics. The table shows point estimates (differences in biomarker levels), 95% confidence interval bounds, p-values, and false-discovery rates (FDR) for interaction terms between the within-batch normalized biomarker level and the potential effect modifier in linear models that have absolute biomarker levels in standard deviation units per biomarker as the outcome and also include main effects for the biomarker and the effect modifier (terms not shown).

| **Biomarker** | **Effect modifier** | **Estimate (95% CI)** | **p** | **FDR** |
| --- | --- | --- | --- | --- |
| Ki-67 | pTNM group | 0.41 (0.20 to 0.63) | 2.0e-04 | 0.012 |
| Beta-catenin | pTNM group | 0.36 (0.00 to 0.71) | 0.047 | 0.629 |
| TUNEL | Calendar year | 0.08 (0.00 to 0.16) | 0.050 | 0.629 |
| SMAD4 | Calendar year | -0.07 (-0.14 to 0.00) | 0.055 | 0.629 |
| Androgen R | pTNM group | 0.11 (-0.01 to 0.23) | 0.066 | 0.629 |
| ER-beta | Calendar year | -0.02 (-0.05 to 0.00) | 0.074 | 0.629 |
| ER-beta | pTNM group | -0.08 (-0.18 to 0.01) | 0.089 | 0.629 |
| Beta-catenin | Calendar year | -0.08 (-0.17 to 0.02) | 0.100 | 0.629 |
| IGF1 R | Gleason score | 0.14 (-0.03 to 0.30) | 0.105 | 0.629 |
| Calcium SR | Calendar year | 0.02 (-0.01 to 0.04) | 0.120 | 0.629 |
| Androgen R | Gleason score | 0.08 (-0.02 to 0.17) | 0.120 | 0.629 |
| Vitamin D R | Calendar year | -0.03 (-0.07 to 0.01) | 0.140 | 0.629 |
| Osteopontin | Calendar year | 0.05 (-0.02 to 0.11) | 0.145 | 0.629 |
| Ki-67 | Calendar year | -0.04 (-0.10 to 0.02) | 0.147 | 0.629 |
| MYC | Calendar year | -0.05 (-0.11 to 0.02) | 0.175 | 0.702 |
| MYC | pTNM group | -0.16 (-0.41 to 0.09) | 0.209 | 0.767 |
| IGF1 R | Calendar year | 0.03 (-0.02 to 0.08) | 0.217 | 0.767 |
| PSMA | Gleason score | -0.25 (-0.66 to 0.17) | 0.247 | 0.797 |
| Insulin R | Calendar year | -0.04 (-0.12 to 0.03) | 0.253 | 0.797 |
| FASN | Calendar year | -0.02 (-0.06 to 0.02) | 0.290 | 0.824 |
| Osteopontin | Gleason score | -0.11 (-0.31 to 0.10) | 0.302 | 0.824 |
| Adiponectin R2 | Gleason score | -0.07 (-0.19 to 0.06) | 0.321 | 0.824 |
| ER-alpha | Gleason score | 0.04 (-0.04 to 0.12) | 0.323 | 0.824 |
| PSMA | Calendar year | 0.07 (-0.08 to 0.23) | 0.352 | 0.824 |
| Adiponectin R2 | Calendar year | -0.02 (-0.06 to 0.02) | 0.352 | 0.824 |
| PTEN-IF | pTNM group | -0.08 (-0.26 to 0.09) | 0.357 | 0.824 |
| Calcium SR | pTNM group | -0.04 (-0.12 to 0.05) | 0.393 | 0.864 |
| PSMA | pTNM group | -0.23 (-0.76 to 0.31) | 0.403 | 0.864 |
| Stathmin-IF | pTNM group | -0.04 (-0.13 to 0.06) | 0.450 | 0.930 |
| FASN | pTNM group | 0.05 (-0.10 to 0.21) | 0.486 | 0.931 |
| Stathmin-IF | Calendar year | 0.01 (-0.01 to 0.03) | 0.504 | 0.931 |
| SMAD4 | pTNM group | 0.09 (-0.18 to 0.36) | 0.521 | 0.931 |
| PTEN-IF | Calendar year | -0.01 (-0.05 to 0.03) | 0.545 | 0.931 |
| Osteopontin | pTNM group | -0.07 (-0.28 to 0.15) | 0.554 | 0.931 |
| Vitamin D R | Gleason score | -0.03 (-0.15 to 0.08) | 0.585 | 0.931 |
| pS6-IF | Gleason score | -0.04 (-0.20 to 0.11) | 0.605 | 0.931 |
| TUNEL | pTNM group | -0.09 (-0.43 to 0.26) | 0.618 | 0.931 |
| pS6-IF | pTNM group | 0.05 (-0.15 to 0.24) | 0.645 | 0.931 |
| MYC | Gleason score | 0.05 (-0.16 to 0.25) | 0.652 | 0.931 |
| FASN | Gleason score | 0.03 (-0.10 to 0.15) | 0.665 | 0.931 |
| SMAD4 | Gleason score | -0.05 (-0.25 to 0.16) | 0.667 | 0.931 |
| IGF1 R | pTNM group | -0.04 (-0.23 to 0.15) | 0.673 | 0.931 |
| Androgen R | Calendar year | 0.01 (-0.02 to 0.04) | 0.676 | 0.931 |
| pS6-IF | Calendar year | -0.01 (-0.05 to 0.03) | 0.729 | 0.931 |
| Cyclin D1 | Calendar year | 0.02 (-0.08 to 0.11) | 0.736 | 0.931 |
| TUNEL | Gleason score | -0.04 (-0.32 to 0.23) | 0.750 | 0.931 |
| Cyclin D1 | Gleason score | 0.05 (-0.28 to 0.37) | 0.775 | 0.931 |
| ER-alpha | pTNM group | -0.01 (-0.11 to 0.08) | 0.777 | 0.931 |
| Insulin R | Gleason score | 0.03 (-0.20 to 0.27) | 0.782 | 0.931 |
| Calcium SR | Gleason score | -0.01 (-0.08 to 0.07) | 0.808 | 0.931 |
| Stathmin-IF | Gleason score | -0.01 (-0.09 to 0.07) | 0.809 | 0.931 |
| ER-beta | Gleason score | 0.01 (-0.07 to 0.09) | 0.828 | 0.931 |
| ER-alpha | Calendar year | 0.00 (-0.02 to 0.02) | 0.836 | 0.931 |
| Adiponectin R2 | pTNM group | 0.02 (-0.14 to 0.17) | 0.838 | 0.931 |
| Insulin R | pTNM group | -0.02 (-0.30 to 0.26) | 0.888 | 0.965 |
| Vitamin D R | pTNM group | -0.01 (-0.15 to 0.14) | 0.929 | 0.965 |
| PTEN-IF | Gleason score | 0.01 (-0.15 to 0.16) | 0.930 | 0.965 |
| Ki-67 | Gleason score | 0.01 (-0.19 to 0.20) | 0.933 | 0.965 |
| Beta-catenin | Gleason score | -0.01 (-0.28 to 0.26) | 0.951 | 0.967 |
| Cyclin D1 | pTNM group | 0.01 (-0.36 to 0.38) | 0.973 | 0.973 |

**Supplementary File 1b.** Intraclass correlation coefficient (ICC) for between-batch variance for uncorrected biomarker levels (“1 Uncorrected”) and biomarker levels after applying different correction methods.

| **Biomarker** | **1 Uncorrected** | **2 Simple mean** | **3 Standardized mean** | **4 IP-weighted mean** | **5 Quantile regression** | **6 Quantile normalization** | **7 ComBat** |
| --- | --- | --- | --- | --- | --- | --- | --- |
| Adiponectin R2 | 0.20 (0.05-0.36) | 0.00 (0.00-0.00) | 0.00 (0.00-0.01) | 0.00 (0.00-0.01) | 0.01 (0.00-0.03) | 0.00 (0.00-0.01) | 0.00 (0.00-0.01) |
| Androgen R | 0.31 (0.09-0.50) | 0.00 (0.00-0.00) | 0.00 (0.00-0.01) | 0.00 (0.00-0.01) | 0.00 (0.00-0.01) | 0.00 (0.00-0.01) | 0.00 (0.00-0.01) |
| Beta-catenin | 0.03 (0.00-0.08) | 0.00 (0.00-0.00) | 0.00 (0.00-0.02) | 0.00 (0.00-0.01) | 0.02 (0.00-0.06) | 0.00 (0.00-0.01) | 0.00 (0.00-0.02) |
| Calcium SR | 0.36 (0.16-0.52) | 0.00 (0.00-0.00) | 0.00 (0.00-0.01) | 0.00 (0.00-0.01) | 0.01 (0.00-0.02) | 0.00 (0.00-0.01) | 0.00 (0.00-0.01) |
| Cyclin D1 | 0.02 (0.00-0.07) | 0.00 (0.00-0.00) | 0.00 (0.00-0.01) | 0.00 (0.00-0.01) | 0.00 (0.00-0.01) | 0.00 (0.00-0.01) | 0.00 (0.00-0.01) |
| ER-alpha | 0.41 (0.19-0.57) | 0.00 (0.00-0.00) | 0.00 (0.00-0.01) | 0.00 (0.00-0.00) | 0.00 (0.00-0.01) | 0.00 (0.00-0.01) | 0.00 (0.00-0.01) |
| ER-beta | 0.37 (0.16-0.53) | 0.00 (0.00-0.00) | 0.00 (0.00-0.01) | 0.00 (0.00-0.01) | 0.00 (0.00-0.01) | 0.00 (0.00-0.01) | 0.00 (0.00-0.01) |
| FASN | 0.18 (0.05-0.33) | 0.00 (0.00-0.00) | 0.00 (0.00-0.01) | 0.00 (0.00-0.01) | 0.00 (0.00-0.01) | 0.00 (0.00-0.01) | 0.00 (0.00-0.01) |
| IGF1 R | 0.13 (0.03-0.26) | 0.00 (0.00-0.00) | 0.00 (0.00-0.01) | 0.00 (0.00-0.01) | 0.03 (0.00-0.08) | 0.00 (0.00-0.01) | 0.00 (0.00-0.01) |
| Insulin R | 0.05 (0.00-0.13) | 0.00 (0.00-0.00) | 0.00 (0.00-0.01) | 0.00 (0.00-0.01) | 0.00 (0.00-0.01) | 0.00 (0.00-0.01) | 0.00 (0.00-0.01) |
| Ki-67 | 0.08 (0.01-0.16) | 0.00 (0.00-0.00) | 0.01 (0.00-0.03) | 0.02 (0.00-0.06) | 0.01 (0.00-0.04) | 0.00 (0.00-0.01) | 0.01 (0.00-0.03) |
| MYC | 0.09 (0.01-0.19) | 0.00 (0.00-0.00) | 0.00 (0.00-0.01) | 0.00 (0.00-0.01) | 0.00 (0.00-0.01) | 0.00 (0.00-0.01) | 0.00 (0.00-0.01) |
| Osteopontin | 0.10 (0.02-0.20) | 0.00 (0.00-0.00) | 0.00 (0.00-0.01) | 0.00 (0.00-0.01) | 0.00 (0.00-0.01) | 0.00 (0.00-0.01) | 0.00 (0.00-0.01) |
| PSMA | 0.01 (0.00-0.03) | 0.00 (0.00-0.00) | 0.00 (0.00-0.01) | 0.00 (0.00-0.01) | 0.00 (0.00-0.01) | 0.00 (0.00-0.01) | 0.00 (0.00-0.01) |
| PTEN-IF | 0.13 (0.04-0.23) | 0.00 (0.00-0.00) | 0.00 (0.00-0.01) | 0.00 (0.00-0.01) | 0.00 (0.00-0.01) | 0.00 (0.00-0.01) | 0.00 (0.00-0.01) |
| SMAD4 | 0.07 (0.01-0.15) | 0.00 (0.00-0.00) | 0.00 (0.00-0.02) | 0.00 (0.00-0.02) | 0.01 (0.00-0.03) | 0.00 (0.00-0.02) | 0.00 (0.00-0.02) |
| Stathmin-IF | 0.48 (0.23-0.63) | 0.00 (0.00-0.00) | 0.00 (0.00-0.01) | 0.00 (0.00-0.01) | 0.01 (0.00-0.03) | 0.00 (0.00-0.01) | 0.00 (0.00-0.01) |
| pS6-IF | 0.08 (0.02-0.15) | 0.00 (0.00-0.00) | 0.00 (0.00-0.01) | 0.00 (0.00-0.01) | 0.00 (0.00-0.01) | 0.00 (0.00-0.01) | 0.00 (0.00-0.01) |
| TUNEL | 0.04 (0.00-0.10) | 0.00 (0.00-0.00) | 0.00 (0.00-0.01) | 0.00 (0.00-0.01) | 0.10 (0.02-0.20) | 0.00 (0.00-0.01) | 0.00 (0.00-0.01) |
| Vitamin D R | 0.24 (0.06-0.41) | 0.00 (0.00-0.00) | 0.00 (0.00-0.01) | 0.00 (0.00-0.01) | 0.00 (0.00-0.01) | 0.00 (0.00-0.01) | 0.00 (0.00-0.01) |

**Supplementary File 1c.** Results from plasmode simulation according to type of induced batch effect, using the data correlation structure “Some confounding.” For three fixed (“true”) hazard ratios for the biomarker–outcome association ($\sqrt{3}$, 1, and 3), the observed hazard ratios after batch correction (with 95% confidence intervals) are shown.

| **True HR** | **Batch effect** | **1 Uncorrected** | **2 Simple mean** | **3 Standardized mean** | **4 IP-weighted mean** | **5 Quantile regression** | **6 Quantile normalization** | **8 IV-pooled** | **9 Strata** |
| --- | --- | --- | --- | --- | --- | --- | --- | --- | --- |
| 1/3 | No batch effects | 0.33 (0.27-0.41) | 0.33 (0.27-0.41) | 0.33 (0.27-0.41) | 0.33 (0.27-0.41) | 0.28 (0.22-0.37) | 0.34 (0.27-0.42) | 0.29 (0.13-0.64) | 0.32 (0.26-0.41) |
| 1/3 | Mean only | 0.46 (0.39-0.55) | 0.33 (0.27-0.41) | 0.33 (0.27-0.41) | 0.33 (0.27-0.41) | 0.34 (0.27-0.42) | 0.34 (0.27-0.42) | 0.29 (0.13-0.64) | 0.32 (0.26-0.41) |
| 1/3 | Mean and variance | 0.44 (0.36-0.54) | 0.37 (0.30-0.45) | 0.37 (0.30-0.45) | 0.37 (0.30-0.45) | 0.34 (0.28-0.43) | 0.32 (0.26-0.41) | 0.32 (0.16-0.64) | 0.34 (0.27-0.44) |
| 1/3 | Effect modification | 0.49 (0.41-0.60) | 0.41 (0.34-0.50) | 0.42 (0.34-0.50) | 0.42 (0.35-0.51) | 0.38 (0.31-0.47) | 0.38 (0.31-0.47) | 0.36 (0.26-0.51) | 0.40 (0.32-0.49) |
| 1 | No batch effects | 0.99 (0.81-1.21) | 0.99 (0.81-1.21) | 0.99 (0.81-1.21) | 0.99 (0.81-1.21) | 0.99 (0.78-1.25) | 0.99 (0.81-1.22) | 0.98 (0.72-1.34) | 0.99 (0.80-1.22) |
| 1 | Mean only | 0.99 (0.85-1.17) | 0.99 (0.81-1.21) | 0.99 (0.81-1.21) | 0.99 (0.81-1.21) | 0.99 (0.81-1.21) | 0.99 (0.81-1.22) | 0.98 (0.72-1.34) | 0.99 (0.80-1.22) |
| 1 | Mean and variance | 0.99 (0.85-1.16) | 0.99 (0.82-1.20) | 0.99 (0.82-1.21) | 0.99 (0.82-1.21) | 0.99 (0.81-1.21) | 0.99 (0.80-1.23) | 0.99 (0.73-1.32) | 0.99 (0.81-1.22) |
| 1 | Effect modification | 1.00 (0.88-1.13) | 0.99 (0.85-1.16) | 0.99 (0.85-1.16) | 0.99 (0.85-1.16) | 0.99 (0.84-1.17) | 1.00 (0.84-1.18) | 1.00 (0.76-1.31) | 0.99 (0.84-1.17) |
| 3 | No batch effects | 3.09 (2.54-3.76) | 3.07 (2.53-3.74) | 3.08 (2.53-3.76) | 3.07 (2.52-3.74) | 3.70 (2.95-4.65) | 3.03 (2.48-3.69) | 3.56 (2.49-5.09) | 3.15 (2.55-3.90) |
| 3 | Mean only | 2.17 (1.86-2.53) | 3.07 (2.53-3.74) | 3.08 (2.53-3.76) | 3.07 (2.52-3.74) | 3.02 (2.49-3.66) | 3.03 (2.48-3.69) | 3.56 (2.49-5.09) | 3.15 (2.55-3.90) |
| 3 | Mean and variance | 1.90 (1.66-2.16) | 2.67 (2.25-3.17) | 2.70 (2.26-3.21) | 2.70 (2.26-3.22) | 2.97 (2.46-3.58) | 3.11 (2.54-3.81) | 3.33 (2.29-4.84) | 3.01 (2.41-3.76) |
| 3 | Effect modification | 1.66 (1.49-1.86) | 2.10 (1.82-2.42) | 2.12 (1.84-2.46) | 2.13 (1.84-2.47) | 2.39 (2.04-2.80) | 2.43 (2.06-2.87) | 3.13 (1.81-5.43) | 2.48 (2.05-2.99) |

**Supplementary File 1d.** Results from plasmode simulation according to data correlation structure, using the batch effect “mean and variance.” For three fixed (“true”) hazard ratios for the biomarker–outcome association ($\sqrt{3}$, 1, and 3), the observed hazard ratios after batch correction (with 95% confidence intervals) are shown.

| **True HR** | **Correlation structure** | **1 Uncorrected** | **2 Simple mean** | **3 Standardized mean** | **4 IP-weighted mean** | **5 Quantile regression** | **6 Quantile normalization** | **8 IV-pooled** | **9 Strata** |
| --- | --- | --- | --- | --- | --- | --- | --- | --- | --- |
| 1/3 | No confounding | 0.43 (0.35-0.53) | 0.37 (0.31-0.46) | 0.37 (0.31-0.46) | 0.38 (0.31-0.46) | 0.40 (0.34-0.49) | 0.32 (0.26-0.40) | 0.31 (0.19-0.50) | 0.34 (0.27-0.43) |
| 1/3 | Some confounding | 0.44 (0.36-0.54) | 0.37 (0.30-0.45) | 0.37 (0.30-0.45) | 0.37 (0.30-0.45) | 0.34 (0.28-0.43) | 0.32 (0.26-0.41) | 0.32 (0.16-0.64) | 0.34 (0.27-0.44) |
| 1/3 | Moderate confounding | 0.45 (0.35-0.56) | 0.37 (0.30-0.46) | 0.37 (0.30-0.46) | 0.37 (0.30-0.46) | 0.30 (0.23-0.39) | 0.33 (0.26-0.42) | 0.32 (0.11-0.91) | 0.35 (0.27-0.45) |
| 1/3 | Confounding+imbalance | 0.44 (0.35-0.55) | 0.38 (0.31-0.47) | 0.37 (0.30-0.46) | 0.39 (0.31-0.48) | 0.30 (0.23-0.39) | 0.33 (0.26-0.41) | 0.32 (0.13-0.76) | 0.34 (0.26-0.45) |
| 1 | No confounding | 1.01 (0.85-1.19) | 1.00 (0.82-1.22) | 1.00 (0.82-1.22) | 1.00 (0.82-1.22) | 1.00 (0.85-1.19) | 1.01 (0.82-1.24) | 1.01 (0.76-1.34) | 1.00 (0.81-1.24) |
| 1 | Some confounding | 0.99 (0.85-1.16) | 0.99 (0.82-1.20) | 0.99 (0.82-1.21) | 0.99 (0.82-1.21) | 0.99 (0.81-1.21) | 0.99 (0.80-1.23) | 0.99 (0.73-1.32) | 0.99 (0.81-1.22) |
| 1 | Moderate confounding | 1.01 (0.87-1.18) | 1.01 (0.84-1.23) | 1.01 (0.84-1.23) | 1.01 (0.84-1.23) | 1.02 (0.82-1.26) | 1.01 (0.83-1.24) | 1.02 (0.75-1.38) | 1.01 (0.82-1.25) |
| 1 | Confounding+imbalance | 1.00 (0.85-1.17) | 1.01 (0.84-1.21) | 1.01 (0.83-1.21) | 1.01 (0.84-1.21) | 1.01 (0.82-1.24) | 1.01 (0.83-1.22) | 1.00 (0.68-1.47) | 1.01 (0.83-1.22) |
| 3 | No confounding | 1.95 (1.68-2.26) | 2.75 (2.29-3.31) | 2.75 (2.29-3.31) | 2.75 (2.29-3.31) | 2.55 (2.15-3.01) | 3.11 (2.53-3.82) | 3.38 (2.25-5.06) | 2.96 (2.41-3.64) |
| 3 | Some confounding | 1.90 (1.66-2.16) | 2.67 (2.25-3.17) | 2.70 (2.26-3.21) | 2.70 (2.26-3.22) | 2.97 (2.46-3.58) | 3.11 (2.54-3.81) | 3.33 (2.29-4.84) | 3.01 (2.41-3.76) |
| 3 | Moderate confounding | 1.82 (1.61-2.07) | 2.54 (2.13-3.03) | 2.60 (2.18-3.11) | 2.61 (2.18-3.12) | 3.36 (2.72-4.15) | 3.04 (2.50-3.69) | 10.78 (4.26-27.3) | 3.04 (2.42-3.82) |
| 3 | Confounding+imbalance | 1.86 (1.65-2.11) | 2.65 (2.22-3.15) | 2.63 (2.21-3.13) | 2.71 (2.27-3.24) | 3.31 (2.68-4.07) | 3.01 (2.46-3.67) | 3.40 (2.17-5.31) | 2.99 (2.40-3.72) |

**Supplementary File 1e.** Gleason grade—biomarker associations according to batch effect correction method. Point estimates from unadjusted linear regression models for biomarker values with Gleason score categories per 1 “grade group” increase as the predictor are shown (with 95% confidence intervals). For *log*_e_-transformed markers like Ki-67, estimates are differences on the loge scale.

| **Marker** | **1 Uncorrected** | **2 Simple mean** | **3 Standardized mean** | **4 IP-weighted mean** | **5 Quantile regression** | **6 Quantile normalization** | **7 ComBat** |
| --- | --- | --- | --- | --- | --- | --- | --- |
| Adiponectin R2 | 0.07 (0.02 to 0.13) | 0.04 (-0.01 to 0.09) | 0.04 (0.00 to 0.09) | 0.04 (-0.01 to 0.09) | 0.04 (0.00 to 0.09) | 0.04 (-0.01 to 0.08) | 0.05 (0.00 to 0.09) |
| Androgen R | 0.03 (-0.03 to 0.08) | 0.01 (-0.04 to 0.05) | 0.01 (-0.04 to 0.05) | 0.01 (-0.04 to 0.05) | 0.00 (-0.05 to 0.05) | 0.01 (-0.03 to 0.05) | 0.01 (-0.04 to 0.05) |
| Beta-catenin | -0.17 (-0.23 to -0.12) | -0.17 (-0.23 to -0.12) | -0.18 (-0.24 to -0.13) | -0.17 (-0.23 to -0.12) | -0.14 (-0.18 to -0.10) | -0.16 (-0.21 to -0.11) | -0.18 (-0.23 to -0.13) |
| Calcium SR | 0.00 (-0.04 to 0.05) | 0.01 (-0.02 to 0.04) | 0.01 (-0.02 to 0.04) | 0.01 (-0.02 to 0.04) | 0.02 (-0.02 to 0.06) | 0.01 (-0.02 to 0.04) | 0.01 (-0.02 to 0.05) |
| Cyclin D1 | 0.06 (0.00 to 0.12) | 0.07 (0.01 to 0.13) | 0.07 (0.01 to 0.13) | 0.07 (0.01 to 0.13) | 0.07 (0.02 to 0.13) | 0.07 (0.01 to 0.12) | 0.07 (0.01 to 0.13) |
| ER-alpha | -0.04 (-0.09 to 0.01) | 0.02 (-0.02 to 0.06) | 0.02 (-0.02 to 0.06) | 0.02 (-0.02 to 0.06) | 0.02 (-0.03 to 0.07) | 0.02 (-0.02 to 0.05) | 0.02 (-0.02 to 0.05) |
| ER-beta | -0.02 (-0.07 to 0.03) | -0.01 (-0.05 to 0.02) | -0.02 (-0.06 to 0.02) | -0.01 (-0.05 to 0.02) | -0.02 (-0.06 to 0.02) | -0.01 (-0.05 to 0.03) | -0.02 (-0.06 to 0.02) |
| FASN | -0.05 (-0.11 to 0.00) | -0.07 (-0.12 to -0.03) | -0.08 (-0.12 to -0.03) | -0.08 (-0.13 to -0.03) | -0.08 (-0.13 to -0.03) | -0.07 (-0.12 to -0.02) | -0.07 (-0.12 to -0.03) |
| IGF1 R | -0.01 (-0.07 to 0.05) | -0.03 (-0.09 to 0.02) | -0.03 (-0.09 to 0.02) | -0.04 (-0.09 to 0.02) | -0.03 (-0.09 to 0.02) | -0.03 (-0.08 to 0.02) | -0.03 (-0.09 to 0.02) |
| Insulin R | 0.04 (-0.02 to 0.10) | 0.02 (-0.04 to 0.08) | 0.02 (-0.04 to 0.08) | 0.02 (-0.04 to 0.08) | 0.02 (-0.03 to 0.08) | 0.02 (-0.04 to 0.08) | 0.02 (-0.04 to 0.08) |
| Ki-67 | 0.10 (0.05 to 0.15) | 0.10 (0.05 to 0.15) | 0.09 (0.04 to 0.15) | 0.09 (0.04 to 0.15) | 0.11 (0.05 to 0.16) | 0.10 (0.05 to 0.16) | 0.09 (0.04 to 0.15) |
| MYC | 0.00 (-0.06 to 0.06) | -0.04 (-0.10 to 0.02) | -0.04 (-0.10 to 0.02) | -0.04 (-0.10 to 0.02) | -0.03 (-0.09 to 0.02) | -0.03 (-0.09 to 0.02) | -0.04 (-0.10 to 0.02) |
| Osteopontin | 0.18 (0.12 to 0.24) | 0.15 (0.10 to 0.21) | 0.16 (0.10 to 0.22) | 0.16 (0.10 to 0.21) | 0.13 (0.08 to 0.19) | 0.14 (0.08 to 0.19) | 0.16 (0.10 to 0.21) |
| PSMA | 0.23 (0.18 to 0.28) | 0.23 (0.17 to 0.28) | 0.23 (0.18 to 0.29) | 0.23 (0.18 to 0.28) | 0.18 (0.14 to 0.22) | 0.23 (0.18 to 0.28) | 0.24 (0.18 to 0.29) |
| PTEN-IF | -0.09 (-0.14 to -0.04) | -0.08 (-0.13 to -0.03) | -0.08 (-0.12 to -0.03) | -0.08 (-0.13 to -0.03) | -0.07 (-0.12 to -0.03) | -0.08 (-0.12 to -0.04) | -0.07 (-0.12 to -0.02) |
| SMAD4 | -0.07 (-0.12 to -0.01) | -0.07 (-0.12 to -0.01) | -0.07 (-0.13 to -0.02) | -0.07 (-0.13 to -0.02) | -0.08 (-0.13 to -0.02) | -0.06 (-0.11 to 0.00) | -0.07 (-0.13 to -0.01) |
| Stathmin-IF | 0.00 (-0.05 to 0.05) | 0.04 (0.00 to 0.07) | 0.04 (0.01 to 0.08) | 0.04 (0.00 to 0.07) | 0.05 (0.01 to 0.09) | 0.04 (0.00 to 0.07) | 0.05 (0.01 to 0.08) |
| pS6-IF | -0.06 (-0.11 to -0.01) | -0.05 (-0.09 to 0.00) | -0.05 (-0.10 to 0.00) | -0.05 (-0.09 to 0.00) | -0.05 (-0.10 to 0.00) | -0.04 (-0.09 to 0.00) | -0.05 (-0.09 to 0.00) |
| TUNEL | -0.01 (-0.07 to 0.06) | 0.01 (-0.05 to 0.07) | 0.01 (-0.05 to 0.07) | 0.01 (-0.04 to 0.07) | -0.01 (-0.25 to 0.23) | 0.01 (-0.05 to 0.06) | 0.01 (-0.05 to 0.07) |
| Vitamin D R | -0.12 (-0.17 to -0.06) | -0.13 (-0.17 to -0.08) | -0.13 (-0.18 to -0.08) | -0.13 (-0.18 to -0.08) | -0.12 (-0.17 to -0.07) | -0.12 (-0.17 to -0.07) | -0.12 (-0.17 to -0.08) |

**Supplementary File 1f.** Biomarker levels and lethal disease according to batch effect correction method. Hazard ratios (with 95% confidence intervals) per 1 standard deviation increase in the biomarker (linear) from unadjusted Cox regression models are shown.

| **Marker** | **1 Uncorrected** | **2 Simple mean** | **3 Standardized mean** | **4 IP-weighted mean** | **5 Quantile regression** | **6 Quantile normalization** | **7 ComBat** |
| --- | --- | --- | --- | --- | --- | --- | --- |
| Adiponectin R2 | 1.33 (1.12-1.59) | 1.49 (1.22-1.82) | 1.49 (1.22-1.82) | 1.49 (1.22-1.81) | 1.57 (1.26-1.95) | 1.57 (1.27-1.95) | 1.51 (1.24-1.85) |
| Androgen R | 1.02 (0.84-1.24) | 0.99 (0.79-1.25) | 0.98 (0.77-1.24) | 0.99 (0.78-1.24) | 0.97 (0.79-1.19) | 0.99 (0.77-1.27) | 0.98 (0.77-1.24) |
| Beta-catenin | 0.84 (0.70-1.01) | 0.82 (0.68-0.99) | 0.80 (0.67-0.97) | 0.82 (0.68-0.99) | 0.74 (0.58-0.94) | 0.82 (0.68-0.99) | 0.80 (0.66-0.97) |
| Calcium SR | 1.17 (1.00-1.37) | 0.97 (0.79-1.21) | 0.98 (0.79-1.21) | 0.97 (0.79-1.21) | 0.95 (0.79-1.14) | 1.00 (0.80-1.26) | 1.02 (0.82-1.26) |
| Cyclin D1 | 1.06 (0.87-1.31) | 1.15 (0.93-1.41) | 1.16 (0.94-1.43) | 1.16 (0.95-1.43) | 1.20 (0.97-1.49) | 1.12 (0.91-1.38) | 1.15 (0.93-1.41) |
| ER-alpha | 0.97 (0.80-1.18) | 0.78 (0.58-1.05) | 0.79 (0.59-1.05) | 0.78 (0.58-1.05) | 0.84 (0.68-1.05) | 0.83 (0.62-1.12) | 0.78 (0.58-1.04) |
| ER-beta | 1.21 (1.03-1.43) | 0.95 (0.75-1.20) | 0.97 (0.77-1.23) | 0.95 (0.75-1.20) | 0.99 (0.78-1.26) | 0.99 (0.78-1.26) | 0.98 (0.77-1.24) |
| FASN | 1.12 (0.92-1.36) | 1.15 (0.92-1.43) | 1.15 (0.92-1.44) | 1.14 (0.91-1.42) | 1.11 (0.90-1.38) | 1.13 (0.90-1.43) | 1.14 (0.91-1.42) |
| IGF1 R | 1.06 (0.85-1.31) | 0.97 (0.77-1.21) | 0.96 (0.76-1.19) | 0.94 (0.76-1.18) | 0.91 (0.74-1.12) | 0.95 (0.74-1.21) | 0.95 (0.76-1.19) |
| Insulin R | 0.94 (0.76-1.16) | 0.92 (0.74-1.14) | 0.92 (0.74-1.14) | 0.90 (0.73-1.13) | 0.89 (0.72-1.11) | 0.93 (0.74-1.16) | 0.91 (0.73-1.13) |
| Ki-67 | 1.51 (1.24-1.84) | 1.63 (1.33-2.01) | 1.58 (1.29-1.93) | 1.59 (1.30-1.94) | 1.50 (1.25-1.81) | 1.61 (1.32-1.97) | 1.56 (1.27-1.91) |
| MYC | 0.95 (0.76-1.20) | 0.92 (0.72-1.17) | 0.91 (0.71-1.17) | 0.92 (0.72-1.17) | 0.86 (0.63-1.16) | 0.88 (0.68-1.14) | 0.91 (0.71-1.16) |
| Osteopontin | 2.45 (1.84-3.26) | 2.08 (1.58-2.72) | 2.12 (1.61-2.79) | 2.04 (1.56-2.67) | 2.11 (1.56-2.86) | 2.22 (1.66-2.97) | 2.15 (1.62-2.85) |
| PSMA | 1.35 (1.12-1.63) | 1.39 (1.15-1.67) | 1.40 (1.16-1.68) | 1.39 (1.15-1.68) | 1.57 (1.22-2.00) | 1.42 (1.17-1.71) | 1.40 (1.16-1.69) |
| PTEN-IF | 0.65 (0.50-0.85) | 0.59 (0.45-0.77) | 0.57 (0.44-0.75) | 0.59 (0.45-0.77) | 0.55 (0.40-0.75) | 0.56 (0.42-0.75) | 0.57 (0.44-0.76) |
| SMAD4 | 1.02 (0.82-1.26) | 1.02 (0.82-1.27) | 1.00 (0.80-1.25) | 1.01 (0.81-1.26) | 0.96 (0.77-1.20) | 1.03 (0.82-1.29) | 1.02 (0.82-1.28) |
| Stathmin-IF | 1.44 (1.21-1.70) | 1.45 (1.13-1.86) | 1.40 (1.09-1.80) | 1.45 (1.13-1.86) | 1.41 (1.11-1.78) | 1.50 (1.15-1.96) | 1.50 (1.16-1.93) |
| pS6-IF | 1.02 (0.83-1.26) | 0.94 (0.76-1.16) | 0.95 (0.77-1.17) | 0.94 (0.76-1.16) | 0.93 (0.75-1.14) | 0.90 (0.72-1.13) | 0.93 (0.75-1.15) |
| TUNEL | 1.05 (0.84-1.31) | 1.03 (0.82-1.30) | 1.04 (0.83-1.31) | 1.04 (0.83-1.31) | 1.02 (0.96-1.08) | 1.06 (0.81-1.39) | 1.04 (0.82-1.31) |
| Vitamin D R | 0.75 (0.60-0.92) | 0.55 (0.42-0.71) | 0.54 (0.42-0.70) | 0.53 (0.41-0.69) | 0.53 (0.41-0.68) | 0.56 (0.43-0.72) | 0.54 (0.42-0.70) |

**Supplementary File 1g.** Biomarker levels and lethal disease according to batch effect correction method. Unlike in the preceding table, the hazard ratios (with 95% confidence intervals) are contrasts comparing extreme quartiles (fourth compared to first quartile) from unadjusted Cox regression models.

| **Marker** | **1 Uncorrected** | **2 Simple mean** | **3 Standardized mean** | **4 IP-weighted mean** | **5 Quantile regression** | **6 Quantile normalization** | **7 ComBat** |
| --- | --- | --- | --- | --- | --- | --- | --- |
| Adiponectin R2 | 1.91 (1.12-3.27) | 2.93 (1.64-5.24) | 2.93 (1.64-5.24) | 3.20 (1.79-5.72) | 2.38 (1.35-4.19) | 2.98 (1.69-5.27) | 2.43 (1.37-4.31) |
| Androgen R | 1.10 (0.61-1.97) | 1.01 (0.58-1.73) | 0.97 (0.56-1.68) | 1.06 (0.61-1.84) | 0.98 (0.57-1.70) | 0.94 (0.55-1.60) | 0.96 (0.56-1.67) |
| Beta-catenin | 0.54 (0.30-0.95) | 0.50 (0.27-0.91) | 0.42 (0.23-0.79) | 0.50 (0.27-0.91) | 0.36 (0.18-0.71) | 0.45 (0.23-0.86) | 0.45 (0.24-0.85) |
| Calcium SR | 1.45 (0.92-2.27) | 1.21 (0.76-1.93) | 1.21 (0.76-1.93) | 1.21 (0.76-1.93) | 0.98 (0.63-1.52) | 1.04 (0.65-1.66) | 1.35 (0.83-2.17) |
| Cyclin D1 | 1.00 (0.58-1.72) | 1.08 (0.63-1.86) | 1.08 (0.62-1.86) | 1.17 (0.68-2.03) | 1.11 (0.65-1.90) | 1.11 (0.65-1.90) | 1.13 (0.65-1.95) |
| ER-alpha | 0.81 (0.47-1.40) | 0.66 (0.37-1.18) | 0.70 (0.40-1.20) | 0.66 (0.37-1.18) | 0.70 (0.41-1.18) | 0.65 (0.38-1.11) | 0.62 (0.35-1.09) |
| ER-beta | 1.46 (0.86-2.47) | 0.82 (0.48-1.39) | 0.94 (0.56-1.58) | 0.82 (0.48-1.39) | 1.01 (0.59-1.73) | 0.96 (0.56-1.63) | 1.05 (0.62-1.78) |
| FASN | 1.27 (0.72-2.24) | 1.13 (0.65-1.98) | 1.18 (0.68-2.04) | 1.04 (0.59-1.84) | 1.03 (0.60-1.78) | 1.11 (0.65-1.92) | 1.12 (0.65-1.91) |
| IGF1 R | 1.11 (0.64-1.94) | 0.83 (0.46-1.47) | 0.83 (0.46-1.47) | 0.82 (0.46-1.46) | 0.92 (0.52-1.62) | 0.85 (0.48-1.51) | 0.83 (0.47-1.48) |
| Insulin R | 0.86 (0.39-1.91) | 0.87 (0.45-1.67) | 0.87 (0.45-1.67) | 0.86 (0.45-1.66) | 0.75 (0.39-1.43) | 0.76 (0.40-1.43) | 0.76 (0.40-1.44) |
| Ki-67 | 2.44 (1.47-4.07) | 3.23 (1.85-5.64) | 2.93 (1.67-5.15) | 2.42 (1.48-3.95) | 2.42 (1.43-4.09) | 2.92 (1.69-5.05) | 2.11 (1.28-3.48) |
| MYC | 0.93 (0.50-1.71) | 1.03 (0.54-1.97) | 0.97 (0.51-1.84) | 1.03 (0.54-1.97) | 0.82 (0.43-1.53) | 0.83 (0.44-1.57) | 0.93 (0.49-1.77) |
| Osteopontin | 5.55 (2.80-10.98) | 5.07 (2.46-10.45) | 4.70 (2.35-9.39) | 4.18 (2.08-8.40) | 4.32 (2.22-8.41) | 4.62 (2.39-8.95) | 4.79 (2.40-9.56) |
| PSMA | 2.20 (1.25-3.86) | 2.65 (1.45-4.83) | 2.24 (1.26-3.99) | 2.63 (1.45-4.80) | 2.34 (1.32-4.15) | 2.44 (1.38-4.33) | 2.49 (1.39-4.49) |
| PTEN-IF | 0.41 (0.22-0.74) | 0.36 (0.19-0.66) | 0.37 (0.20-0.69) | 0.36 (0.19-0.66) | 0.31 (0.16-0.60) | 0.33 (0.18-0.62) | 0.32 (0.17-0.60) |
| SMAD4 | 1.05 (0.59-1.84) | 0.80 (0.46-1.40) | 0.69 (0.39-1.23) | 0.91 (0.51-1.61) | 0.95 (0.52-1.71) | 0.91 (0.51-1.64) | 0.75 (0.43-1.31) |
| Stathmin-IF | 1.85 (1.04-3.28) | 2.11 (1.20-3.72) | 1.75 (1.00-3.05) | 2.11 (1.20-3.72) | 1.63 (0.91-2.90) | 1.80 (1.02-3.18) | 1.64 (0.94-2.84) |
| pS6-IF | 1.25 (0.71-2.18) | 0.86 (0.48-1.56) | 0.91 (0.51-1.62) | 0.86 (0.48-1.56) | 0.84 (0.48-1.47) | 0.84 (0.48-1.47) | 1.01 (0.57-1.79) |
| TUNEL | 1.11 (0.59-2.07) | 1.13 (0.61-2.10) | 1.17 (0.64-2.16) | 1.16 (0.63-2.14) | 0.94 (0.49-1.81) | 1.14 (0.62-2.13) | 1.53 (0.81-2.88) |
| Vitamin D R | 0.44 (0.23-0.86) | 0.19 (0.09-0.41) | 0.19 (0.09-0.42) | 0.19 (0.09-0.40) | 0.17 (0.08-0.37) | 0.19 (0.09-0.40) | 0.20 (0.09-0.43) |
