# Supplementary material for: Extent, impact, and mitigation of batch effects in tumor biomarker studies using tissue microarrays
Source: eLife. 2021 Dec 23;10:e71265. doi: 10.7554/eLife.71265 (PMC8849344; doi:10.7554/eLife.71265)
Supplement: Source code 1. [file elife-71265-code1.zip › batchtma_manuscript_211206.html]

Extent, impact, and mitigation of batch effects in tumor biomarker studies using tissue microarrays


Code 

- Show All Code
- Hide All Code

# Extent, impact, and mitigation of batch effects in tumor biomarker studies using tissue microarrays

#### Konrad H. Stopsack stopsack@post.harvard.edu

#### Mon Dec 06, 2021

```
# Bootstrap repeats, used for all ICC calculations
bootrepeat <- 500

Sys.setenv("TZ" = "EST5EDT")  # workaround to avoid Sys.timezone() error in library()
# https://github.com/rocker-org/rocker-versioned/issues/89

library(here)      # here() for relative paths
library(batchtma)  # install with remotes::install.github("stopsack/batchtma")
library(khsverse)  # with khsmisc; remotes::install.github("stopsack/khsverse")

# Used but not attached:
# * library(FactoMineR)  # PCA(); principal components analysis
# * library(ggrepel)     # geom_text_repel() for Figure 4--S2 (supplement 2)
# * library(grid)        # load PNG images for Fig. 3
# * library(png)         # load PNG images for Fig. 3, continued
# * library(survminer)   # ggsurvplot(); Kaplan-Meier curves in Figure 5--S1
# * library(rms)         # splines in Fig. 6
# * library(Hmisc)       # cut2()
# * library(lme4)        # lmer(); fit mixed models for custom ICC estimation
# * library(rptR)        # rpt() for ICCs
# * library(quantreg)    # for analyses of het_quantiles (Fig. 4--S5)
# * library(broom.mixed) # tidy.merMod() for mixed models
# * library(sva)         # ComBat(); from Bioconductor

theme_set(theme_cowplot())
```

```
# Load preprocessed datasets and output from simulation study
# a) Biomarker list
markers <- readRDS(file = here("data", "preprocessed", "biomarkers_211203.rds"))

# b) Cohort and biomarker data
comb <- readRDS(file = here("data", "preprocessed", "combined_data_210309.rds"))

# c) Core-level estrogen receptor data
core_er <- readRDS(file = here("data", "preprocessed", "cores_er_210310.rds"))

# d) Simulation data
# Hazard ratios for Fig. 5 DEF
allhrs <- read_csv(file = here("out", "sim_allhrs_500_210309.csv")) %>%
  mutate(
    batcheff = factor(batcheff, levels = 1:4,
                      labels = c("No batch effects", "Mean only",
                                 "Mean and variance", "Effect modification")),
    datalabel = factor(datalabel, levels = 1:4,
                       labels = c("No confounding", "Some confounding",
                                  "Moderate confounding", "Confounding+imbalance")))

# Data structure for Suppl. Fig. 10
dfsim2 <- readRDS(file = here("out", "sim_data_df2_500_210309.rds"))

#############################################################################################

# Perform log transformation for select biomarkers
# and z scale all biomarkers
comb <- comb %>% 
  mutate_at(.vars = vars(markers %>% filter(trans == "log") %>% pull(var)), 
            .funs = list(log = ~log(. + abs(min(c(-0.0001, .), na.rm = TRUE))))) %>%
  mutate_at(.vars = vars(markers$var_use),
            .funs = ~as.numeric(scale(.)))

markers <- markers %>%
  mutate(tumors = map_int(.x = var_use,
                          .f = ~sum(!is.na(comb %>% select(all_of(.x))))),
         tmas   = map_int(.x = var_use,
                          .f = ~comb %>% 
                            filter(!is.na(get(.x))) %>% 
                            pull(tma) %>%
                            unique(.) %>%
                            length(.)),
         tma_ids = map_chr(.x = var_use,
                           .f = ~comb %>% filter(!is.na(get(.x))) %>% 
                             arrange(tma) %>%
                             pull(tma) %>%
                             unique(.) %>%
                             paste(., collapse = " ")))

#############################################################################################
# Perform batch adjustments

model_linetypes <- c(
  "1 Uncorrected"            = "solid", 
  "2 Simple mean"            = "dashed",
  "3 Standardized mean"      = "dotted",
  "4 IP-weighted mean"       = "dotdash",
  "5 Quantile regression"    = "twodash",
  "6 Quantile normalization" = "dotted",
  "7 ComBat"                 = "longdash",
  "8 IV-pooled"              = "longdash",
  "9 Strata"                 = "dotdash")
model_labels    <- names(model_linetypes)   # names only
model_linetypes <- unname(model_linetypes)  # values only

# Add corrected values for approaches 2-6, using batchtma::adjust_batch()
comb <- comb %>% 
  adjust_batch(markers = markers$var_use, batch = tma, method = simple) %>%
  adjust_batch(markers = markers$var_use, batch = tma, method = standardize,
               confounders = c(dxyear, gleason, ptnm_miss2)) %>%
  adjust_batch(markers = markers$var_use, batch = tma, method = ipw,
               confounders = c(dxyear, gleason, ptnm_miss2)) %>%
  adjust_batch(markers = markers$var_use[markers$var_use != "mean_casr_intensity"], 
               batch = tma, method = quantreg,
               confounders = c(dxyear, gleason, ptnm_miss2),
               quantreg_tau = c(0.25, 0.75),
               quantreg_method = "fn") %>%
  adjust_batch(markers = mean_casr_intensity,  # too much collinearity for method = "fn"
               batch = tma, method = quantreg,
               confounders = c(dxyear, gleason, ptnm_miss2),
               quantreg_tau = c(0.25, 0.75),
               quantreg_method = "br") %>%
  adjust_batch(markers = markers$var_use, batch = tma, method = quantnorm)

# Approach 7: ComBat
call_combat <- function(data, markers) {
  tmas <- data %>%
    select(id, tma, all_of(markers)) %>%
    pivot_longer(cols = c(-id, -tma), names_to = "marker", values_to = "value") %>%
    group_by(tma, marker) %>%
    summarize(nonmiss = sum(!is.na(value)), .groups = "drop") %>%
    group_by(tma) %>%
    summarize(markers = sum(nonmiss > 0), .groups = "drop") %>%
    ungroup() %>%
    filter(markers > 0) %>%
    pull(tma)
  df <- data %>% 
    filter(tma %in% tmas)
  
  expre <- df %>%
    select_at(.vars = vars(all_of(markers))) %>%
    as.matrix() %>% 
    t()
  pheno <- df %>%
    select(gleason, ptnm_miss2, dxyear, tma)
  modelcombat <- model.matrix(~ gleason + ptnm_miss2 + dxyear, data = pheno)
  combat_edata <- sva::ComBat(dat = expre, batch = pheno$tma, 
                              mod = modelcombat, 
                              par.prior = TRUE, prior.plots = FALSE) %>%
    t() %>% 
    as_tibble() %>% 
    rename_all(.funs = ~paste0(., "_adj7")) %>%
    bind_cols(x = df %>% select(id), y = .) %>%
    left_join(x = data, y = ., by = "id")
}

comb <- comb %>%
  call_combat(markers = markers %>% filter(tmas == 8)  %>% pull(var_use)) %>%
  call_combat(markers = markers %>% filter(tmas == 9)  %>% pull(var_use)) %>%
  call_combat(markers = markers %>% filter(tmas == 10) %>% pull(var_use)) %>%
  call_combat(markers = markers %>% filter(tmas == 14) %>% pull(var_use))
```

# Figure 1

**Characteristics of men with prostate cancer with tissue included on the 14 tumor tissue microarrays.** **A**, Calendar years of cancer diagnosis, with thick lines indicating median, boxes interquartile ranges, and whiskers 1.5 times the interquartile range. **B**, Counts of tumors by Gleason score. **C**, Counts of tumors by pathological TNM stage (RP: radical prostatectomy). **D**, Rates of lethal disease (metastases or prostate cancer-specific death over long-term follow-up), with bars indicating 95% confidence intervals.

```
combplot <- comb %>% 
  mutate(tma = fct_rev(factor(tma)),
         ptnm = fct_collapse(ptnm_miss, 
                             "No RP" = c("missing", "M1"),
                             "pT3b+" = c("pT3b", "pT4/N1")))
part1 <- combplot %>%
  ggplot(mapping = aes(y = dxyear, x = tma)) +
  geom_boxplot(outlier.shape = NA) +
  coord_flip() +
  theme(panel.grid.major.x = element_line(color = "gray90"),
        axis.line.y = element_blank(),
        axis.ticks.y = element_blank(),
        axis.text.y = element_text(hjust = 0.5)) +
  labs(x = "Tissue microarray",
       y = "Year of cancer diagnosis")

part2 <- combplot %>% 
  ggplot(mapping = aes(fill = gleason, x = tma)) + 
  geom_bar(position = position_stack(reverse = TRUE), stat = "count") +
  scale_fill_viridis_d(option = "cividis") +
  scale_y_continuous(expand = c(0, 0)) +
  labs(x = "Tissue microarray", y = "Proportion", fill = "Gleason") +
  coord_flip() +
  guides(fill = guide_legend(nrow = 2, byrow = TRUE)) +
  theme(legend.position = "bottom",
        panel.grid.major.x = element_line(color = "gray90"),
        axis.ticks.y = element_blank(),
        axis.text.y  = element_blank(),
        axis.title   = element_blank(),
        axis.line.y  = element_blank(),
        legend.spacing.x = unit(0.05, "cm"),
        legend.spacing.y = unit(0.1, "cm"),
        legend.title = element_text(vjust = 1),
        legend.margin = margin(0, 0, 0, 0),
        legend.box.margin = margin(-10, -10, -10, -10))

part3 <- combplot %>% 
  ggplot(mapping = aes(fill = ptnm, x = tma)) + 
  geom_bar(position = position_stack(reverse = TRUE), stat = "count") +
  scale_fill_viridis_d(option = "cividis") +
  scale_y_continuous(expand = c(0, 0)) +
  labs(x = "Tissue microarray", y = "Proportion", fill = "pTNM") +
  coord_flip() +
  guides(fill = guide_legend(nrow = 2, byrow = TRUE)) +
  theme(legend.position = "bottom",
        panel.grid.major.x = element_line(color = "gray90"),
        axis.ticks.y = element_blank(),
        axis.text.y  = element_blank(),
        axis.title   = element_blank(),
        axis.line.y  = element_blank(),
        legend.spacing.x = unit(0.05, "cm"),
        legend.spacing.y = unit(0.1, "cm"),
        legend.title = element_text(vjust = 1),
        legend.margin = margin(0, 0, 0, 0),
        legend.box.margin = margin(-10, -10, -10, -10))


part4 <- combplot %>%
  group_by(tma) %>%
  summarize(events      = sum(eventlethal),
            personyears = sum(lethalfu) / 12,
            ir          = events / personyears,  
            # Use Poisson approximation for variance of incidence rate:
            ll          = exp(log(ir) - qnorm(0.975) * 1 / sqrt(events)),
            ul          = exp(log(ir) + qnorm(0.975) * 1 / sqrt(events)),
            ir_form     = round(ir, digits = 0),
            .groups     = "drop") %>%
  mutate_at(.vars = vars(ir, ll, ul), .funs = ~.*1000) %>%
  ggplot(mapping = aes(x = ir, xmin = ll, xmax = ul, 
                       y = tma, label = ir_form)) +
  geom_errorbarh(size = 0.5, height = 0) +
  geom_point(size = 3) +
  coord_cartesian(clip = "off") +
  scale_x_continuous(expand = c(0, 0), 
                     breaks = seq(from = 5, to = 20, by = 5)) +
  labs(x = "Lethal events per\n1000 person-years", y = "") +
  theme(panel.grid.major.x = element_line(color = "gray90"),
        axis.ticks.y = element_blank(),
        axis.text.y  = element_blank(),
        axis.title.y = element_blank(),
        axis.line.y  = element_blank())

plot_grid(part1, part2, part3, part4, 
          labels = LETTERS, label_x = -0.002, label_y = 1.008, hjust = 0,
          nrow = 1, align = "h", axis = "tlbr")
```

# Figure 2

**Biomarkers stained, staining and scoring methods, and intraclass correlation coefficients (ICCs).** **A**, Tissue microarrays assessed for each marker (dark blue, yes). **B**, Approach to staining biomarkers: automated staining system vs. manual staining (gray, yes); quantification of biomarkers: software-based scoring vs. eye scoring (blue, yes); biomarker quality assessed: staining intensity, proportion of cells positive for the biomarker, area of tissue positive for the biomarker (yellow, yes). **C**, Counts of tumors assessed for each biomarker. **D**, Between-tissue microarray ICCs (i.e., proportion of variance explained by between-tissue microarray differences) for each biomarker, with 95% confidence intervals. Empty symbols indicate the 97.5th percentile of the null distribution of the ICC obtained by permuting tumor assignments to tissue microarrays; asterisks indicate between-Gleason grade group ICCs. Biomarkers are arranged by descending between-tissue microarray ICC.

```
# ICCs based on lmer() with bootstrapped CIs
# Need to suppress bootstrapping progress with 'results = "hide"' in markdown header
icc_oneway <- function(marker, data, righthand = "~ 1 + (1 | tma)", group = "tma") {
  set.seed(123)
  res <- rptR::rpt(formula = as.formula(paste(marker, righthand)),
                   grname   = group, 
                   data     = data %>%  # exclude -Inf for log-transformed markers
                     filter(!is.na(get(marker)) & get(marker) != -Inf) %>%  
                     mutate(tma = factor(tma)),
                   datatype = "Gaussian", 
                   nboot    = bootrepeat,  # in global environment
                   npermut  = 0, 
                   parallel = FALSE)  # edited with shell submission
  bind_cols(res$CI_emp %>% as_tibble(rownames = "term"), 
            ICC = res$R %>% t() %>% as.numeric()) %>%
    select(ICC, ll = `2.5%`, ul = `97.5%`)
}

# Null value for ICC based on permuting samples between TMAs samples 
icc_null <- function(marker, data) {
  set.seed(123)  # testdata has different lengths per biomarker;
                 # so it is probably okay to re-set the seed each time
  testdata <- data %>% 
    select(tma, marker = one_of(marker)) %>%
    filter(!is.na(marker) & marker != -Inf) %>%
    mutate(tma = factor(tma))
  tibble(index = 1:bootrepeat) %>%
    mutate(
      data = map(.x = index, .f = ~{
        testdata %>%  # shuffle TMA assigments for individual tumors:
          mutate(tma = tma[sample.int(n = nrow(testdata))])}),
      icc = map_dbl(.x = data, .f = ~{
        summary(lme4::lmer(formula = marker ~ 1 + (1 | tma), 
                           data = .x)) %>%
          pluck("varcor") %>%
          as_tibble() %>%
          mutate(icc = vcov / sum(vcov)) %>%
          slice(1) %>%
          pull(icc)
      })) %>%
    pull(icc) %>% 
    quantile(., probs = 0.975)
}


markersfig2 <- markers %>%
  select(var_use, name, tumors, staining, scoring, int, cells, area) %>%
  mutate(res = map(.x = var_use, .f = icc_oneway, 
                   data = comb)) %>%
  unnest_wider(col = res) %>%
  arrange(desc(ICC)) %>%
  mutate(name      = fct_inorder(name),
         icc_round = format(round(ICC, digits = 2), nsmall = 2),
         icc_null  = map_dbl(.x = var_use, .f = icc_null, data = comb))

icc_gleason <- markers %>%
  select(var_use, name) %>%
  mutate(res = map(.x = var_use, 
                   .f = icc_oneway, 
                   data = comb, 
                   righthand = "~ (1 | gleason)", 
                   group = "gleason")) %>%
  unnest_wider(col = res)
```

```
part1 <- markersfig2 %>%
  expand_grid(tma = unique(comb$tma)) %>%
  mutate(exists = map2_dbl(.x = var_use, .y = tma,
                           .f = ~{
                             comb %>% 
                               filter(!is.na(get(.x))) %>% 
                               filter(tma == .y) %>%
                               nrow() }),
         exists = if_else(exists > 0, TRUE, FALSE)) %>%
  ggplot(mapping = aes(x = factor(tma), 
                       y = fct_rev(name), 
                       fill = fct_rev(factor(exists)))) +
  geom_tile(color = "white", size = 0.3) +
  scale_fill_manual(values = c(viridis_pal(option = "cividis")(10)[1],
                               "gray80")) +
  scale_x_discrete(expand = c(0, 0)) +
  scale_y_discrete(expand = c(0, 0)) +
  labs(x = "Tissue microarray") +
  theme(
    axis.title.y = element_blank(),
    panel.grid.major = element_blank(),
    panel.background = element_blank(),
    axis.line = element_blank(),
    axis.ticks = element_blank(),
    legend.position = "none",
    legend.justification = c(1, 0),
    legend.direction = "vertical",
    axis.text.x = element_text(vjust = 0.7, hjust = 0.5))

part2 <- markersfig2 %>%
  mutate(Automated = if_else(staining == "Automated", 1, 0),
         Manual    = if_else(staining == "Manual",    1, 0),
         Software  = if_else(scoring == "Software",   2, 0),
         Eye       = if_else(scoring == "Eye",        2, 0),
         Intensity = if_else(int == TRUE,             3, 0),
         `% Cells` = if_else(cells == TRUE,           3, 0),
         Area      = if_else(area == TRUE,            3, 0)) %>%
  select(name, Automated:Area) %>%
  pivot_longer(cols = -name, names_to = "attribute", values_to = "value") %>%
  mutate(attribute = fct_inorder(factor(attribute))) %>%
  ggplot(mapping = aes(x = attribute, y = fct_rev(name), 
                       fill = factor(value))) +
    geom_tile(color = "white", size = 0.3) +
    scale_fill_manual(values = c(
      "gray80",
      "gray30",
      viridis_pal(option = "cividis")(10)[3],
      viridis_pal(option = "cividis")(10)[8])) +
    scale_x_discrete(expand = c(0, 0)) +
    scale_y_discrete(expand = c(0, 0)) +
    theme(
      axis.title.x = element_blank(),
      axis.title.y = element_blank(),
      panel.grid.major = element_blank(),
      panel.background = element_blank(),
      axis.line = element_blank(),
      axis.ticks = element_blank(),
      legend.position = "none",
      legend.justification = c(1, 0),
      legend.direction = "vertical",
      axis.text.y = element_blank(),
      axis.text.x = element_text(angle = 45, vjust = 1, hjust = 1))

part3 <- markersfig2 %>%
  ggplot(mapping = aes(x = fct_rev(name), y = tumors)) +
  geom_bar(stat = "identity") +
  scale_y_continuous(expand = c(0, 0), 
                     breaks = seq(from = 0, to = 1000, by = 500)) +
  labs(x = "", y = "Tumors (n)") +
  coord_flip() +
  theme(panel.grid.major.x = element_line(color = "gray85"),
        axis.text.y = element_blank(),
        axis.ticks.y = element_blank(),
        axis.title.y = element_blank(),
        axis.line.y = element_blank())

part4 <- markersfig2 %>%
  left_join(icc_gleason %>% select(var_use, icc_gleason = ICC), by = "var_use") %>%
  ggplot(mapping = aes(x = ICC, xmin = ll, xmax = ul, 
                       y = fct_rev(name), label = icc_round)) +
  geom_errorbarh(size = 0.5, height = 0) +
  geom_point(size = 3) +
  geom_text(nudge_y = 0.45, size = 3) +
  geom_point(mapping = aes(x = icc_null), shape = 0) +
  geom_point(mapping = aes(x = icc_gleason), shape = 8) +
  scale_x_continuous(limits = c(0, 0.7), expand = c(0, 0),
                     breaks = seq(from = 0, to = 6, by = 0.1)) +
  labs(x = "ICC:\nBetween-batch variance / total variance", y = "") +
  coord_cartesian(clip = "off") +
  theme(panel.grid.major.x = element_line(color = "gray90"),
        axis.line.y = element_blank(),
        axis.text.y = element_blank(),
        axis.ticks.y = element_blank(),
        axis.title.y = element_blank(),
        plot.margin = margin(t = 0.4, unit = "cm"))

plot_grid(part1, part2, part3, part4, 
          rel_widths = c(1, 0.38, 0.4, 1),
          labels = LETTERS, label_x = -0.012, label_y = 1.01, 
          nrow = 1, align = "h", axis = "tlbr")
```

## Figure Supplement 1

Tissue microarrays and differences in % positivity, at the example of estrogen receptor alpha and beta, and variance in biomarker levels explained by between-tissue microarray differences (ICC).

```
er_positivity_icc <- tribble(
  ~var_use,                ~name,
  "log_er_alpha_positive", "ER alpha: % cells positive (log)",
  "cub_er_beta_positive",  "ER beta: % cells positive (cubic)") %>%
  mutate(res = map(.x = var_use, .f = icc_oneway, 
                   data = comb %>% 
                     # normality transformtions, as for intensity values
                     mutate(log_er_alpha_positive = log(er_alpha_positive + 
                                                          abs(min(c(-0.0001, 
                                                                    er_alpha_positive),
                                                                  na.rm = TRUE))),
                            cub_er_beta_positive = er_beta_positive^3))) %>%
  unnest_wider(col = res) %>%
  mutate_if(.predicate = is.numeric,
            .funs      = ~format(round(., digits = 2), nsmall = 2))

icc_alpha <- er_positivity_icc %>% filter(var_use == "log_er_alpha_positive")
icc_alpha <- paste0(icc_alpha["ICC"], "; 95% CI, ", icc_alpha["ll"], " to ", icc_alpha["ul"])
icc_beta <- er_positivity_icc %>% filter(var_use == "cub_er_beta_positive")
icc_beta <- paste0(icc_beta["ICC"], "; 95% CI, ", icc_beta["ll"], " to ", icc_beta["ul"])

plot_grid(stripplot(data = comb %>% mutate(tma = factor(tma)), 
                    x = tma, y = er_alpha_positive) + 
                    labs(x = "TMA", y = "ER alpha: % cells positive",
                    title = paste0("Estrogen receptor alpha positivity: ICC, ", icc_alpha)) +
            theme(plot.title = element_text(size = 12, face = "bold", hjust = 0),
                  panel.grid.major.y = element_line(color = "gray90")),
          stripplot(data = comb %>% mutate(tma = factor(tma)), 
                    x = tma, y = er_beta_positive) + 
                    labs(x = "TMA", y = "ER beta: % cells positive",
                    title = paste0("Estrogen receptor beta positivity: ICC, ", icc_beta)) +
            theme(plot.title = element_text(size = 12, face = "bold", hjust = 0),
                  panel.grid.major.y = element_line(color = "gray90")),
          ncol = 1, labels = LETTERS)
```

# Figure 3

**Effect of batch effect mitigation on a biomarker with strong between-tissue microarray variation.** **A**, The protein biomarker estrogen receptor-alpha was quantified as staining intensity in nuclei of epithelial cells, averaged over all cores of each tumor. Each panel shows processed data for a specific approach to correcting batch effects. Notes in the upper right corner indicate which properties of batch effects were potentially addressed. Each data point represents one tumor. y-axes are standard deviations of the combined data for the specific method. Thick lines indicate medians, boxes interquartile ranges, and whisker length is 1.5 times the interquartile range. **B**, Example photographs of TMAs; brown color indicates positive staining.

```
stripplot_local <- function(..., title, xtitle, ytitle) {
  stripplot(...) +
    labs(title = title, x = xtitle, y = ytitle)
}
# _adj6 for IGF1-R is missing--catch this
pos_stripplot <- possibly(.f = stripplot_local, 
                          otherwise = ggdraw() + draw_label(label = "(missing)"))
  
marker_by_tma <- function(biomarker, label, describemethods = FALSE, extra = NULL) {
  model_character <- c("1" = "None", 
                       "2" = "Means", 
                       "3" = "Standardized means", 
                       "4" = "IP-weighted means", 
                       "5" = "First quartile, adjusted\nInterquartile range, adjusted",
                       "6" = "Means\nVariance\nRank distances",
                       "7" = "Adjusted means\n(Variance)\n(Rank distances)")
  
  panels <- map(.x = 1:7, .f = ~{
    myplot <- pos_stripplot(data = comb %>% mutate(tma = factor(tma)), x = tma, 
                            y = paste0(biomarker, if_else(condition = .x > 1, 
                                                          true = paste0("_adj", .x), 
                                                          false = "")), 
                            title = model_labels[.x], ytitle = "Biomarker [SDs]",
                            xtitle = "Tissue microarray") +
      theme(plot.title = element_text(size = 12, face = "bold", hjust = 0),
            panel.grid.major.y = element_line(color = "gray85"))  +
      scale_y_continuous(breaks = -1:5, limits = c(-2, 7))
    if(describemethods == TRUE) {
      myplot <- myplot +
        labs(tag = paste("Corrected:", model_character[as.character(.x)])) +
        theme(plot.tag.position = c(.97, 1),
              plot.tag = element_text(hjust = 1, size = unit(10, "pt"), 
                                      face = "plain")) 
    }
    if(.x %in% c(1:3, 5:6))
      myplot <- myplot + theme(axis.title.x = element_blank(),
                               axis.text.x = element_blank())
    if(.x %in% c(5:7))
      myplot <- myplot + theme(axis.title.y = element_blank())
    myplot 
  })

  if(label != "")
    plot_grid(
      ggdraw() +
        draw_label(label = label, fontface = "bold", x = 0, hjust = 0) +
        theme(plot.margin = margin(0, 0, 0, 7)),
      plot_grid(
        plot_grid(plotlist = panels[c(1, 5, 2, 6)],
                  ncol = 2, align = "hv", axis = "bt"),
        plot_grid(plotlist = panels[c(3, 7, 4)],
                  ncol = 2, align = "hv", axis = "bt"),
        nrow = 2, ncol = 1, rel_heigths = c(0.4, 0.5)),
      ncol = 1, rel_heights = c(0.05, 1))
  else
    plot_grid(
      plot_grid(plotlist = panels[c(1, 5, 2, 6)],
                ncol = 2, align = "hv", axis = "bt", labels = "A"),
      plot_grid(plotlist = append(panels[c(3, 7, 4)], list(extra)),
                ncol = 2, align = "hv", axis = "bt", labels = c("", "", "", "B")),
      nrow = 2, ncol = 1, rel_heights = c(0.4, 0.5))
    
}


tmaimage <- function(tma) {
  ggdraw(grid::rasterGrob(png::readPNG(here("data", "thumbnails", 
                                            paste0("thumb", tma, ".png"))),
                          interpolate = TRUE)) +
    geom_text(data = data.frame(x = 0.5, y = 0.1, label = tma),
              aes(x, y, label = label), hjust = 0.5,
              inherit.aes = FALSE)
}

part2 <- plot_grid(tmaimage("A"), tmaimage("H"), tmaimage("K"), tmaimage("N"),
                   nrow = 1)

marker_by_tma(biomarker = "er_alpha_intens", label = "", 
              describemethods = TRUE, extra = part2)
```

## Figure Supplement 1

Uncorrected compared with batch effect-corrected biomarker levels, for estrogen receptor alpha. Symbols and color indicate the tissue microarray.

```
scatteradj <- function(marker, title) {
  myplot <- comb %>%
    select(starts_with(marker), tma) %>%
    rename(unadj = one_of(marker)) %>%
    pivot_longer(cols = c(-unadj, -tma), names_to = "adj", values_to = "val") %>%
    mutate(tma = factor(tma),
           adj = str_remove(string = adj, pattern = marker),
           adj = str_remove(string = adj, pattern = "_adj"),
           adj = factor(adj, levels = 2:7,
                        labels = model_labels[2:7])) %>%
    ggplot(mapping = aes(x = unadj, y = val, color = tma, shape = tma)) +
    geom_abline(linetype = "dashed", color = "black") +
    geom_point() +
    facet_wrap(~ adj) +
    scale_color_viridis_d(name = "Tissue\nmicroarray", option = "cividis") +
    scale_shape_manual(name = "Tissue\nmicroarray", values = 0:20) +
    theme(panel.grid.major = element_line(color = "gray90"),
          strip.background = element_rect(fill = "transparent"), 
          strip.text = element_text(face = "bold")) +
    labs(x = "Uncorrected biomarker levels", y = "Batch-corrected biomarker levels", 
         title = title) +
    coord_fixed()
  print(myplot)
}

scatteradj(marker = "er_alpha_intens", title = "")
```

# Figure 4

**Patterns, source, and remediation of batch effects.** **A**, Biomarker mean levels by tissue microarray, in standard deviations per biomarker (y-axis). Each point is one tissue microarray. **B**, First two principal components of biomarkers quantified on all 14 tissue microarrays, with symbol color/shape by tissue microarray. Each point is one tumor. **C**, The same first two principal components, with color/shape by Gleason score. **D**, Per-core biomarker levels for tumors with cores included on two separate tissue microarrays per tumor, for estrogen receptor (ER) alpha and beta, both in standard deviations. Each point is one tumor core. **E**, Pearson correlation coefficients *r* between uncorrected biomarker levels (bottom row) and biomarkers after corrections, showing the mean r across all markers.

```
# For panel 1 (and later use): mean per TMA
tmamean <- comb %>%
  group_by(tma) %>%
  summarize_at(.vars = vars(markers$var_use),
               .funs = mean, na.rm = TRUE) %>%
  mutate_at(.vars = vars(-tma), 
            .funs = ~. - mean(., na.rm = TRUE))

# For panel 2 (and later use): PCA wrapper, can subset to markers or TMAs
mypca <- function(data, plotmarkers, colorshape = "tma", legend = "TMA") {
  df <- data %>% 
    select_at(.vars = vars(tma, gleason, one_of(plotmarkers))) %>%
    mutate_at(.vars = vars(one_of(plotmarkers)),
              .funs = list(nana = ~!is.na(.))) %>%
    mutate(nonmiss = pmap_int(.l = select(., ends_with("_nana")),
                              .f = sum)) %>%
    filter(nonmiss > 0)
  
  df_pca <- df %>%
    select_at(.vars = vars(one_of(plotmarkers))) %>%
    FactoMineR::PCA(graph = FALSE)  # does mean imputation for missing values
  df$pc1_new <- df_pca$ind$coord[, 1]   # First PC
  df$pc2_new <- df_pca$ind$coord[, 2]   # Second PC
  
  df %>%
    ggplot(mapping = aes(x = pc1_new, y = pc2_new, 
                         color = factor(get(colorshape)), 
                         shape = factor(get(colorshape)))) +
    geom_point(size = 2) +
    scale_shape_manual(name = legend, values = 20:0) +
    scale_colour_viridis_d(name = legend, option = "cividis") +
    labs(x = "Principal component 1", y = "Principal component 2")
}

set.seed(123)
part1 <- tmamean %>%
  pivot_longer(cols = c(-tma), names_to = "marker", values_to = "mean") %>%
  left_join(markers %>% select(var_use, name), by = c("marker" = "var_use")) %>%
  ggplot(mapping = aes(x = factor(tma), y = mean, color = name, shape = name)) +
  geom_hline(yintercept = 0, linetype = "dashed") +
  geom_jitter(height = 0, width = 0.2, size = 3) +
  labs(x = "Tissue microarray", y = "Difference to grand mean") +
  guides(color = guide_legend(ncol = 2, byrow = FALSE)) +
  scale_color_viridis_d(name = "Biomarker", option = "cividis") +
  scale_shape_manual(name = "Biomarker", values = 0:20) +
  scale_y_continuous(breaks = seq(from = -1, to = 1.5, by = 0.5)) +
  theme(panel.grid.major.y = element_line(color = "gray85"),
        panel.grid.major.x = element_line(color = "gray85"))

part2 <- mypca(data = comb, legend = "Tissue\nmicroarray",
               plotmarkers = markers %>% filter(tmas > 9) %>% pull(var_use))

part3 <- mypca(data = comb,
               plotmarkers = markers %>% filter(tmas > 9) %>% pull(var_use),
               colorshape = "gleason", legend = "Gleason\nscore")

set.seed(123)
part4 <- core_er %>%
  mutate(marker = case_when(marker == "ERalpha" ~ "ER-alpha", 
                            marker == "ERbeta"  ~ "ER-beta")) %>%
  filter(nuc_mean_z < 7) %>%  # exclude one very extreme value (z score of 8)
  ggplot(mapping = aes(x = as.factor(as.numeric(factor(id))), y = nuc_mean_z)) + 
  geom_jitter(mapping = aes(color = factor(tma), shape = factor(tma)),
              height = 0, width = 0.1, size = 3) +
  scale_color_viridis_d(name = "Tissue\nmicroarray", option = "cividis") +
  scale_shape_manual(name = "Tissue\nmicroarray", values = 15:35) +
  labs(x = "Participant (tumor)", y = "Biomarker level per tumor core") + 
  theme(panel.grid.major = element_line(color = "gray85"),
        strip.background = element_rect(fill = "transparent"), 
        strip.text = element_text(face = "bold")) +
  facet_wrap(~ marker, scales = "free")

################################################################################
# Generate correlation matrix
corrmat_data <- function(data, method = "pearson", reorder = TRUE) {
  # matrix reorder by hierarchical clustering
  reorder_cormat <- function(cormat) {
    # Use correlation between variables as distance
    dd <- as.dist((1-cormat)/2)
    hc <- hclust(dd)
    cormat <- cormat[hc$order, hc$order]
  }
  
  # Get upper triangle of the correlation matrix
  get_upper_triangle <- function(cormat){
    cormat[lower.tri(cormat)]<- NA
    return(cormat)
  }
  
  mymat <- data %>%
    cor(method = method)
  if(reorder == TRUE)
    mymat <- reorder_cormat(mymat)
  mymat %>%
    get_upper_triangle() %>%
    as_tibble(rownames = "var1") %>% 
    pivot_longer(cols = -var1, names_to = "var2", values_to = "value", 
                 values_drop_na = TRUE)
}

# Plot correlation matrix
corrmat_plot <- function(data, method, cutpoints = c(-1, 0, 1), digits = 2) {
  data %>%
    group_by(var1) %>%
    mutate(ylabel = if_else(row_number() == 1, 
                            true = as.character(var1), false = "")) %>%
    ungroup() %>%
    ggplot(mapping = aes(var2, var1, fill = value))+
    geom_tile(color = "white")+
    scale_fill_gradient2(
      low      = viridis_pal(option = "cividis")(20)[1], 
      high     = viridis_pal(option = "cividis")(20)[16], 
      mid      = viridis_pal(option = "cividis")(20)[8], 
      midpoint = cutpoints[2], 
      limit    = c(cutpoints[1], cutpoints[3]), 
      space    = "Lab", 
      name     = paste(str_to_title(method), "correlation", sep = "\n")) +
    coord_fixed(clip = "off") +
    geom_text(aes(x = var2, y = var1, 
                  label = format(round(value, digits = digits), nsmall = digits)), 
              color = "black", size = 4) +
    geom_text(mapping = aes(x = as.numeric(var2) - 0.7, 
                            y = as.numeric(var1), 
                            label = ylabel),
              hjust = 1) +
    scale_x_discrete(expand = c(0, 0)) +
    scale_y_discrete(expand = c(0, 0)) +
    theme(
      axis.title.x = element_blank(),
      axis.title.y = element_blank(),
      axis.text.x = element_text(angle = 45, vjust = 1, 
                                 size = 12, hjust = 1),
      axis.text.y = element_blank(),
      panel.grid.major = element_blank(),
      panel.border = element_blank(),
      panel.background = element_blank(),
      axis.line = element_blank(),
      axis.ticks = element_blank(),
      legend.justification = c(1, 0),
      legend.position = c(-0.35, 0.35),
      plot.margin = margin(t = 0, r = 0, b = 0, l = 2, unit = "cm"),
      legend.direction = "vertical") +
    guides(fill = guide_colorbar(barwidth = 1, barheight = 5,
                                 title.position = "top", title.hjust = 0.5))
}

# Parallelize for all markers: 
#   1) Generate correlation matrixes
#   2) Summarize correlation coefficients as mean
#   3) Plot
corrmat_all <- function(data, method, ...) {
  markers$var_use %>%
    map_dfr(.f = ~{
      comb %>% 
        select(starts_with(.x)) %>%
        filter(!is.na(get(.x))) %>%
        rename_at(.vars = vars(all_of(.x)), .funs = ~paste0(.x, "_adj1")) %>%
        corrmat_data(method = method, reorder = FALSE) %>%   
        mutate(marker = .x, # this does not work with mutate_at() for some reason
               var1 = str_remove(string = var1, pattern = paste0(.x, "_adj")),
               var2 = str_remove(string = var2, pattern = paste0(.x, "_adj"))) %>%
        mutate_at(.vars = vars(var1, var2), .funs = as.numeric)
    }) %>%
    group_by(var1, var2) %>%
    summarize(value = median(value), .groups = "drop") %>%
    mutate_at(.vars = vars(var1, var2),
              .funs = ~fct_inorder(factor(., levels = 1:7, 
                                          labels = model_labels[1:7]))) %>%
    corrmat_plot(method = method, ...)
}


part5 <- corrmat_all(method = "pearson", cutpoints = c(0.85, 0.925, 1))

left_plots <- align_plots(part1, part2, part4, align = "v", axis = "l")
plot_grid(left_plots[[1]],
          plot_grid(left_plots[[2]], part3, 
                    ncol = 2, align = "hv", labels = c("B", "C")),
          plot_grid(left_plots[[3]], part5, 
                    ncol = 2, labels = c("D", "E")),
          ncol = 1, labels = "A")
```

## Figure Supplement 1

Ratios of variance per tissue microarray to the mean variance for each marker.

```
set.seed(123)
comb %>%
  group_by(tma) %>%
  summarize_at(.vars = vars(markers$var_use),
               .funs = var, na.rm = TRUE) %>%
  mutate_at(.vars = vars(-tma), .funs = ~. / mean(., na.rm = TRUE)) %>%
  pivot_longer(cols = c(-tma), names_to = "marker", values_to = "var") %>%
  left_join(markers %>% select(var_use, name), by = c("marker" = "var_use")) %>%
  ggplot(mapping = aes(x = factor(tma), y = var, color = name, shape = name)) +
  geom_hline(yintercept = 1, linetype = "dashed") +
  geom_jitter(height = 0, width = 0.2, size = 3) +
  labs(x = "Tissue microarray", y = "Ratio of variance") +
  guides(color = guide_legend(ncol = 2, byrow = FALSE)) +
  scale_color_viridis_d(name = "Biomarker", option = "cividis") +
  scale_shape_manual(name = "Biomarker", values = 0:20) +
  scale_y_continuous(breaks = seq(from = -1, to = 3, by = 0.5)) +
  theme(panel.grid.major.y = element_line(color = "gray85"),
        panel.grid.major.x = element_line(color = "gray85"))
```

## Figure Supplement 2

Intraclass correlation coefficients (ICCs), quantifying the proportion of variance in biomarker levels attributable to between-tissue microarray differences, across all tumors and after restriction to those 378 tumors across tissue microarrays that have the same clinical/pathological characteristics in terms of Gleason score 3+4 and prostatectomy stage pT1/T2.

```
icc_restrict <- markers %>%
  select(var_use, name) %>%
  mutate(res = map(.x = var_use, .f = icc_oneway, 
                   data = comb %>% 
                     filter(gleason == "3+4" & 
                              # Assume that non-missing pTNM implies "RP"
                              ptnm_miss2 == "pT1/T2"))) %>%
  unnest_wider(col = res)
```

```
bind_rows(markersfig2 %>% 
            mutate(ICC_copy = ICC), 
          icc_restrict, .id = "type") %>%
  group_by(name) %>%
  mutate(ICC_copy = median(ICC_copy, na.rm = TRUE)) %>%
  ungroup() %>%
  arrange(ICC_copy, type) %>%
  mutate(type  = factor(type, levels = 1:2, 
                       labels = c("All", "Restricted: Gleason 3+4, pT1/T2")),
         icc_round = format(round(ICC, digits = 2), nsmall = 2),
         name  = as.numeric(fct_inorder(factor(name))),
         ypos  = name + if_else(type == "All", 0.13, -0.13),
         ypos2 = name + if_else(type == "All", 0.45, -0.45)) %>%
  ggplot(mapping = aes(x = ICC, xmin = ll, xmax = ul, 
                       y = ypos, label = icc_round,
                       color = type)) +
  geom_errorbarh(size = 0.5, height = 0) +
  geom_point(size = 3) +
  ggrepel::geom_text_repel(mapping = aes(y = ypos2), direction = "x", 
                  segment.alpha = 0,  # suppress line between label and point
                  point.padding = NA) +  # to not move away from dot, only from other text
  coord_cartesian(clip = "off") +
  scale_color_viridis_d(name = "Tumors", option = "cividis", end = 0.8) +
  scale_x_continuous(limits = c(0, 1), 
                     expand = c(0, 0),
                     breaks = seq(from = 0, to = 1, by = 0.1)) +
  scale_y_continuous(breaks = 1:nlevels(factor(markersfig2$name)),
                     labels = levels(fct_rev(factor(markersfig2$name))),
                     expand = c(0.02, 0.02)) +
  labs(x = "ICC (between-batch variance / total variance)", y = "") +
  theme(panel.grid.major.x = element_line(color = "gray85"),
        axis.line.y = element_blank(),
        axis.ticks.y = element_blank(),
        axis.text.y = element_text(margin = margin(r = 0.5, unit = "cm")),
        legend.position = c(0.4, 0.2),
        legend.background = element_rect(color = "black", fill = "white"),
        legend.margin = margin(6, 6, 6, 6))
```

## Figure Supplement 3

Intraclass correlation coefficients (ICCs), quantifying the proportion of variance in biomarker levels attributable to between-Gleason grade differences, by increasing ICC.

```
ploticc <- function(data) {
  data %>%
    arrange(ICC) %>%
    mutate(name = fct_inorder(name),
           icc_round = format(round(ICC, digits = 2), nsmall = 2)) %>%
    ggplot(mapping = aes(x = ICC, xmin = ll, xmax = ul, 
                         y = fct_rev(name), label = icc_round)) +
    geom_errorbarh(size = 0.5, height = 0) +
    geom_point(size = 3) +
    geom_text(nudge_y = 0.4) +
    coord_cartesian(clip = "off") +
    scale_x_continuous(limits = c(0, 1), 
                       expand = c(0, 0),
                       breaks = seq(from = 0, to = 1, by = 0.1)) +
    scale_y_discrete(expand = c(0.01, 0.01)) +
    labs(x = "ICC (between-batch variance / total variance)", y = "") +
    theme(panel.grid.major.x = element_line(color = "gray85"),
          axis.line.y = element_blank(),
          axis.ticks.y = element_blank(),
          axis.text.y = element_text(margin = margin(r = 0.5, unit = "cm")))
}

icc_gleason %>% ploticc()
```

## Figure Supplement 4

Principal components 1 and 2 after batch effect correction using (7) quantile normalization for biomarkers available on all tissue microarrays. Symbol color and shape indicate the tissue microarray.

```
mypca(data = comb,
      plotmarkers = paste0(markers %>% 
                             filter(tmas == 14) %>%  # new markers on all TMAs
                             pull(var_use), "_adj7"))
```

## Figure Supplement 5

Quantile-specific associations of confounders (clinical/pathological differences) with (uncorrected) biomarker levels of estrogen receptor alpha. Shown are regression coefficients for the 10th, 50th, and 90th percentiles as the outcomes of quantile regression models.

```
batchrq_confounders <- function(data, variable, confounders, batch = "tma") {
  data <- data %>% 
    filter(!is.na(get(variable))) %>%
    mutate(batchvar = factor(get(batch)))
  
  quantreg::rq(formula = as.formula(paste(variable, "~ batchvar", confounders)), 
               data = data, 
     tau = c(0.1, 0.5, 0.9)) %>%
    tidy() %>%
    filter(term != "(Intercept)") %>%
    filter(!str_detect(string = term, pattern = "batchvar"))
}

het_quantiles <- function(marker, title) {
  myplot <- batchrq_confounders(data = comb %>% mutate(dxyear_10y = dxyear/10), 
                                variable = marker, 
                                confounders = " + gleason + ptnm_miss2 + dxyear_10y") %>%
    mutate(quantile = factor(tau)) %>%
    mutate(term = recode(term,
                         "gleason3+4"        = "Gleason score 3+4",
                         "gleason4+3"        = "Gleason score 4+3",
                         "gleason8"          = "Gleason score 8",
                         "gleason9-10"       = "Gleason score 9-10",
                         "ptnm_miss2pT3/T3a" = "pTNM pT3",
                         "ptnm_miss2pT3b"    = "pTNM pT3b",
                         "ptnm_miss2hirisk"  = "pTNM T4/N1/no RP",
                         "dxyear_10y"        = "Calendar year (decades)")) %>%
    ggplot(mapping = aes(x = fct_rev(factor(term)), y = estimate, 
                         ymin = conf.low, ymax = conf.high, 
                         color = quantile)) +
    geom_hline(yintercept = 0, linetype = "dashed") +
    geom_errorbar(position = position_dodge(width = 0.5), size = 0.5, width = 0.2) +
    geom_point(position = position_dodge(width = 0.5), size = 3) +
    labs(y = "Coefficient [standard deviations]", x = "", title = title) +
    theme(panel.grid.major.x = element_line(color = "gray85"),
          panel.grid.minor.x = element_line(color = "gray95")) +
    scale_color_viridis_d(name = "Quantile", option = "cividis", end = 0.8) +
    coord_flip()
  print(myplot)
}

het_quantiles(marker = "er_alpha_intens", title = "")
```

## Figure Supplement 6

Batch corrections per tissue microarray and method. The plot shows the difference between uncorrected and corrected values per batch, averaged across all biomarkers. For batch correction approaches that only address the mean (i.e., that subtract the same correction value from all biomarker values within each batch), only that difference is visible; for correction methods that address individual values within batches differently, batch-specific medians and interquartile ranges of differences between uncorrected and corrected values are visible.

```
set.seed(123)
comb %>%
  select(id, tma, starts_with(markers$var_use)) %>%
  pivot_longer(cols = c(-id, -tma), names_to = "var", values_to = "val") %>%
  mutate(
    marker = str_remove_all(string = var, pattern = "_adj[1-9]"),
    adjustment = str_remove_all(string = var, 
                         pattern = paste(markers$var_use, collapse = "|")),
    adjustment = as.numeric(str_remove_all(string = adjustment, pattern = "_adj")),
    raw = if_else(is.na(adjustment), val, NA_real_)) %>%
  group_by(id, tma, marker) %>%
  mutate(raw = median(raw, na.rm = TRUE)) %>%
  ungroup() %>%
  mutate(diff = raw - val) %>%
  group_by(tma, marker, adjustment) %>%
  summarize(median = median(diff, na.rm = TRUE),
            q25    = quantile(diff, probs = 0.25, na.rm = TRUE),
            q75    = quantile(diff, probs = 0.75, na.rm = TRUE),
            .groups = "drop") %>%
  group_by(tma, adjustment) %>%
  summarize(median = mean(median, na.rm = TRUE),
            q25    = mean(q25, probs = 0.25, na.rm = TRUE),
            q75    = mean(q75, probs = 0.75, na.rm = TRUE),
            .groups = "drop") %>%
  filter(!is.na(adjustment)) %>%
  mutate(tma = factor(tma),
         type = factor(adjustment, levels = c(2:7),
                       labels = model_labels[2:7])) %>%
  ggplot(mapping = aes(x = tma, y = median, 
                       ymin = q25, ymax = q75, 
                       color = type, shape = type, group = type)) +
  geom_pointrange(position = position_dodge(width = 0.8)) +
  scale_color_viridis_d(name = "", option = "cividis") +
  scale_shape(name = "") +
  labs(x = "Tissue microarray", y = "Difference [SDs], median (interquartile range)") +
  guides(color = guide_legend(ncol = 2, byrow = FALSE)) +
  theme(panel.grid.major = element_line(color = "gray85"),
        legend.key.width = unit(1.5, "cm"),
        axis.text = element_text(size = 10),
        axis.title = element_text(size = 10),
        legend.position = "bottom")
```

## Figure Supplement 7

Biomarker differences in ER-alpha intensity, after batch effect correction methods, for a one-unit increment in Gleason score, stratified by tissue microarray. “Pooled” indicates estimates pooled over batches (TMAs) using inverse-variance weighting. “No stratification” indicates estimates without stratification.

```
data_gleason_by_tma <- function(marker) {
  comb %>%
    select(tma, starts_with(marker)) %>%
    pivot_longer(cols = -tma, names_to = "adjust", values_to = "val") %>%
    filter(!is.na(val)) %>%
    count(tma, adjust) %>%
    select(-n) %>%
    mutate(adj = str_remove(string = adjust, pattern = marker),
           adj = as.numeric(str_remove(string = adj, pattern = "_adj")),
           adj = if_else(is.na(adj), 1, adj)) %>%
    mutate(model = map2(.x = tma, .y = adjust,
                        .f = ~lm(
                          formula = as.formula(paste0(.y, 
                                                      " ~ as.numeric(gleason)")), 
                          data = comb %>% filter(tma == .x))),
           res = map(.x = model, .f = ~{tidy(.x, conf.int = TRUE) %>% slice(2)})) %>%
    unnest(cols = res) %>%
    select(tma, adj, estimate, std.error, conf.low, conf.high) %>%
    mutate(adj = factor(adj, levels = 1:length(model_labels),
                        labels = model_labels),
           tma = factor(tma))
}

ivpooled <- function(data) {
  data %>% 
    mutate(weight = 1 / (std.error ^ 2),
           tma = as.character(tma)) %>%
    group_by(adj) %>%
    summarize(
      tma       = "Pooled",
      estimate  = sum(estimate * weight) / sum(weight),
      std.error = sqrt(1 / sum(weight)),
      conf.low  = estimate - qnorm(0.975) * std.error,
      conf.high = estimate + qnorm(0.975) * std.error,
      .groups = "drop")
}

gleason_marginal <- function(marker) {
  comb %>%
    select(starts_with(marker)) %>%
    pivot_longer(cols = everything(), names_to = "adjust", values_to = "val") %>%
    filter(!is.na(val)) %>%
    count(adjust) %>%
    select(-n) %>%
    mutate(adj = str_remove(string = adjust, pattern = marker),
           adj = as.numeric(str_remove(string = adj, pattern = "_adj")),
           adj = if_else(is.na(adj), 1, adj)) %>%
    mutate(model = map(.x = adjust,
                        .f = ~lm(
                          formula = as.formula(paste(.x, 
                                                     "~ as.numeric(gleason)")), 
                          data = comb)),
           res = map(.x = model, .f = ~{ tidy(.x, conf.int = TRUE) %>% slice(2) })) %>%
    unnest(cols = res) %>%
    select(adj, estimate, std.error, conf.low, conf.high) %>%
    mutate(adj = factor(adj, levels = 1:length(model_labels),
                        labels = model_labels),
           tma = "No\nstratification")
}

stratified_plot <- function(data, title) {
  myplot <- data %>%
    ggplot(mapping = aes(x = tma, y = estimate, 
                         ymin = conf.low, ymax = conf.high, 
                         color = adj, shape = adj)) +
    geom_hline(yintercept = 0, color = "gray50", linetype = "dashed") +
    geom_pointrange(position = position_dodge(width = 0.8)) +
    scale_color_viridis_d(name = "Method", option = "cividis") +
    scale_shape_manual(name = "Method", values = c(15:18, 8, 12, 5, 6)) +
    labs(x = "Tissue microarray", y = "Beta per 1 Gleason score (in SDs, with 95% CI)",
         title = title) +
    theme(panel.grid.major = element_line(color = "gray85"))
  print(myplot)
}

gleason_forest <- function(marker, title) {
  data <- data_gleason_by_tma(marker = marker)
  bind_rows(data %>% ivpooled(),
            data %>% mutate(tma = as.character(tma)),
            gleason_marginal(marker = marker)) %>%
    mutate(tma = factor(tma, levels = c(levels(factor(comb$tma)), 
                                        "Pooled", "No\nstratification"))) %>%
    stratified_plot(title = title)
}

gleason_forest(marker = "er_alpha_intens", title = "")
```

# Figure 5

**Plasmode simulation results.** **A**–**C**, Biomarker levels by tissue microarray in three simulation scenarios; **D**–**F**, true versus observed hazard ratios for the biomarker–outcome association after alternative approaches to batch effect correction. The solid blue line indicates no correction for batch effects. **A** and **D**, no batch effects; **B** and **E**, means-only batch effects; **C** and **F**, means and variance batch effects.

```
# For main figure, use scenario (2): "Some confounding"
# Biomarker values forced to be associated with Gleason and stage (modestly)
set.seed(123)
dfexp2 <- comb %>%
  select(id, tma, gleason, ptnm_miss2, eventlethal, lethalfu) %>%
  filter(lethalfu != 0) %>%
  mutate(exposure   = rnorm(n = nrow(.)),
         # must collapse stage pT3 because of non-convergence of flexsurvreg() in dfexp4
         ptnm_miss2 = fct_collapse(ptnm_miss2, pT3 = c("pT3/T3a", "pT3b")),
         exposure = exposure + 
           0.2 * (as.numeric(gleason)    - mean(as.numeric(gleason))) +
           0.1 * (as.numeric(ptnm_miss2) - mean(as.numeric(ptnm_miss2))))

all_adjustments <- function(data, markers, drop = FALSE) {
  res <- data %>% 
    adjust_batch(markers = all_of(markers), batch = tma, method = simple) %>%
    adjust_batch(markers = all_of(markers), batch = tma, method = standardize,
                 confounders = c(gleason, ptnm_miss2)) %>%
    adjust_batch(markers = all_of(markers), batch = tma, method = ipw,
                 confounders = c(gleason, ptnm_miss2)) %>%
    adjust_batch(markers = all_of(markers), batch = tma, method = quantreg,
                 confounders = c(gleason, ptnm_miss2), quantreg_tau = c(0.25, 0.75)) %>%
    adjust_batch(markers = all_of(markers), batch = tma, method = quantnorm)
  if(drop == TRUE)
    res <- res %>% select(id, starts_with(markers), tma)
  res
}

# Add batch effects
add_batcheffects <- function(realdata) {
  # as per real data
  tma_means_er_alpha <- c("A"  = 1.23, "B"  = 0.71, "C" = 0.25, "D" = 0.69,
                          "E" = 0.54, "F" = 0.4, "G" = -0.06, "H" = -0.71, 
                          "I" = -0.34, "J" = -0.74, "K" = -0.98, "L" = -0.35, 
                          "M" = -0.14, "N" = -0.5)
  tma_vars_er_alpha  <- c("A"  = 1.48, "B"  = 1.1, "C"  = 1.02, "D" = 1.16,
                          "E" = 2.31, "F" = 1.22, "G" = 1.09, "H" = 0.25,
                          "I" = 0.7, "J" = 0.45, "K" = 0.57, "L" = 0.67, 
                          "M" = 1.09, "N" = 0.89)  
  realdata %>% 
    # (B) means only, like for ER-alpha
    mutate(exposureBbtc = exposure + tma_means_er_alpha[as.character(tma)]) %>%  
    group_by(tma) %>%
    # (C) means and variance, like for ER-alpha
    mutate(exposureCbtc = (exposure - mean(exposure)) *
             sqrt(tma_vars_er_alpha[as.character(tma)]) +
             mean(exposure) + tma_means_er_alpha[as.character(tma)],
           # (D) means, variance like for ER-alpha plus effect modification by Gleason
           exposureDbtc = (exposure - mean(exposure)) * 
             sqrt(tma_vars_er_alpha[as.character(tma)]) * 
             (as.numeric(gleason) - mean(as.numeric(gleason)) + 6)/6 +
             mean(exposure) + 
             tma_means_er_alpha[as.character(tma)] * (as.numeric(gleason) - 
                                                        mean(as.numeric(gleason)) + 6)/6) %>%
    ungroup() %>%
    all_adjustments(markers = "exposure") %>%
    all_adjustments(markers = "exposureBbtc") %>%
    all_adjustments(markers = "exposureCbtc") %>%
    all_adjustments(markers = "exposureDbtc")
}

dfexp2 <- dfexp2 %>% add_batcheffects()

# Plotting function for observed vs. expected hazard ratios
plot_adj_hrs <- function(data) {
  if(!("datalabel" %in% names(data)))
    data <- data %>% mutate(datalabel =  "", batcheff = "")
  data <- data %>%
    select(truehr, starts_with("hr_"), datalabel, batcheff) %>%
    pivot_longer(cols = c(-truehr, -datalabel, -batcheff), 
                 names_to = "adjust", values_to = "obshr") %>%
    mutate(adjust = as.numeric(str_remove(string = adjust, pattern = "hr_adj")),
           adjust = factor(adjust, levels = 1:length(model_labels), 
                           labels = model_labels)) %>%
    group_by(datalabel, batcheff, truehr, adjust) %>%
    summarize(yval = median(obshr, na.rm = TRUE), .groups = "drop")
  myplot <- data %>%
    ggplot(mapping = aes(x = truehr, y = yval, 
                         color = adjust, group = adjust, linetype = adjust)) +
    coord_fixed(clip = "off") +
    geom_abline(linetype = "dashed") +
    geom_path(size = 1) +
    scale_x_continuous(expand = c(0.001, 0), limits = c(0.33, 3),
                       breaks = c(0.33, 0.5, 1, 2, 3), 
                       labels = c("0.33", "0.5", "1", "2", "3"),
                       trans = "log") +
    scale_y_continuous(expand = c(0.001, 0), limits = c(0.28, 3.5),
                       breaks = c(0.33, 0.5, 1, 2, 3),
                       labels = c("0.33", "0.5", "1", "2", "3"),
                       trans = "log") +
    scale_color_viridis_d(name = "", option = "cividis") +
    scale_linetype_manual(name = "", breaks = model_labels,
                          values = model_linetypes) +
    theme(panel.grid.major = element_line(color = "gray85")) +
    labs(x = "Expected hazard ratio", 
         y = "Observed hazard ratio", 
         color = "", linetype = "")
  if("datalabel" %in% names(data)) {
    myplot +
      guides(color = guide_legend(nrow = 2, byrow = TRUE)) +
      theme(legend.position = "bottom")
  }
  else {
    myplot +
      guides(color = guide_legend(ncol = 3, byrow = TRUE))  +
      theme(legend.position = "top")
  }
}


# Dot plots of biomarkers by batch
stripplot_batch <- function(exposurevar, title) {
  myicc <- icc_oneway(marker = exposurevar, data = dfexp2) %>% 
    as.numeric() %>% 
    round(., digits = 2) %>%
    format(., nsmall = 2)
  
  dfexp2 %>% 
    mutate(tma = factor(tma)) %>%
    stripplot(x = tma, y = one_of(exposurevar), printplot = FALSE) +
    scale_y_continuous(limits = c(min(c(dfexp2$exposure, 
                                        dfexp2$exposureBbtc, dfexp2$exposureCbtc)),
                                  max(c(dfexp2$exposure, 
                                        dfexp2$exposureBbtc, dfexp2$exposureCbtc))),
                       breaks = seq(from = -4, to = 4, by = 1)) +
    theme(plot.title = element_text(size = 12, face = "bold"),
          panel.grid.major.y = element_line(color = "gray90")) +
    labs(title = title, x = "Tissue microarray", y = "Biomarker [SDs]",
         subtitle = paste0("ICC, ", myicc[1], " (95% CI, ", 
                           myicc[2], " to ", myicc[3], ")"))
}

# Observed vs. expected hazard ratios
part4 <- allhrs %>%
  filter(batcheff == "No batch effects") %>%
  filter(datalabel == "Some confounding") %>%
  plot_adj_hrs() + theme(legend.key.width = unit(2, "cm"), 
                         legend.justification = "center")
part5 <- allhrs %>%
  filter(batcheff == "Mean only") %>%
  filter(datalabel == "Some confounding") %>%
  plot_adj_hrs() +
  theme(axis.title.y = element_blank())
part6 <- allhrs %>%
  filter(batcheff == "Mean and variance") %>%
  filter(datalabel == "Some confounding") %>%
  plot_adj_hrs() +
  theme(axis.title.y = element_blank())

plot_grid(
  plot_grid(stripplot_batch(exposurevar = "exposure",     
                            title = "No batch effects"), 
            stripplot_batch(exposurevar = "exposureBbtc", 
                            title = "Batch effects for mean only") +
              theme(axis.title.y = element_blank()), 
            stripplot_batch(exposurevar = "exposureCbtc", 
                            title = "Batch effects for mean and variance") +
              theme(axis.title.y = element_blank()), 
            part4 + theme(legend.position = "none"), 
            part5 + theme(legend.position = "none"), 
            part6 + theme(legend.position = "none"),
            ncol = 3, labels = LETTERS, align = "hv", axis = "tlbr"),
  get_legend(part4),
  nrow = 2, ncol = 1, rel_heights = c(0.9, 0.1))
```

## Figure Supplement 1

Data structures in the actual data and in 200 plasmode simulation datasets. **A**, Gleason scores and lethal prostate cancer (metastasis-free survival) in the actual data. **B**, Gleason scores and lethal prostate cancer in an example simulated dataset. Shaded areas indicate 95% confidence intervals. **C**, Pearson correlation coefficients between biomarker levels and confounders, and between confounders, across all simulated datasets. Correlation coefficients observed in the actual data are noted in the legend. **D**, Hazard ratios for the biomarker and the confounders in relation to lethal prostate cancer, pooling all simulated data sets. Confounder–outcome associations were simulated to correspond to their observed values in the actual data; exposure–outcome associations were simulated to a range of hazard ratios (*x* axis). Lines indicate medians across simulations with the same exposure–outcome hazard ratio, shaded areas range from the 2.5th to 97.5th percentile.

```
# Kaplan-Meier curves in original and simulated data
plotkm <- function(data, variable, title = "") {
  myexposure <- data %>% 
    select(myexposure = one_of(variable)) %>% 
    pull(myexposure)
  myformula <- as.formula(paste("Surv(time = lethalfu/12, event = eventlethal) ~",
                                variable))
  fit <- survfit(formula = myformula, data = data)
  fit$call$formula <- myformula
  fit$call$data <- data
  
  survminer::ggsurvplot(
    fit = fit,
    data = data,
    conf.int      = TRUE,
    risk.table    = FALSE,
    palette       = viridis_pal(option = "cividis")(length(levels(myexposure))),
    ylab          = "Metastasis-free survival",
    xlab          = "Time since cancer diagnosis [years]",
    legend.labs   = levels(myexposure),
    ylim          = c(0.5, 1),
    break.time.by = 4,
    surv.scale    = "percent",
    legend.title = title) +
    guides(color = guide_legend(nrow = 1))
}
part1 <- dfexp2 %>%
  plotkm(variable = "gleason", title = "Gleason")
part2 <- dfsim2 %>% slice(2) %>% pull(data) %>% pluck(1) %>%
  plotkm(variable = "gleason", title = "Gleason")

# Plot true exposure-outcome hazard ratio vs. observed exposure-outcome and 
# confounder-outcome hazard ratios
plot_true_vs_obs_hrs <- function(df, predictors = "exposure + gleason + ptnm_miss2", 
                                 rename_list = NULL) {
  df %>%
    mutate(model  = map(.x = data, 
                        .f = coxph, 
                        formula = as.formula(paste(
                          "Surv(time = eventtime, event = status) ~ ",
                          predictors))),
           coefs  = map(.x = model, .f = tidy, exponentiate = TRUE)) %>%
    select(coefs, truehr) %>%
    unnest(cols = coefs) %>%
    mutate(term = recode(.x = term, !!!rename_list)) %>%
    group_by(term, truehr) %>%
    summarize(median = median(estimate),
              ll     = quantile(estimate, probs = 0.025),
              ul     = quantile(estimate, probs = 0.975),
              .groups = "drop") %>%
    mutate(ul = if_else(ul > 10,  10,  ul),
           ll = if_else(ll < 0.1, 0.1, ll)) %>%
    ggplot(mapping = aes(x = truehr, y = median, ymin = ll, ymax = ul, group = term)) +
    geom_abline(linetype = "dashed") +
    geom_line(mapping = aes(color = term, linetype = term), size = 1) +
    geom_ribbon(alpha = 0.1) +
    scale_y_continuous(trans = "log", #limits = c(0.3, 3),
                       breaks = c(0.33, 0.5, 1, 2, 3), expand = c(0, 0)) +
    scale_x_continuous(trans = "log", 
                       limits = c(0.33, 3),
                       breaks = c(0.33, 0.5, 1, 2, 3), expand = c(0, 0)) +
    theme(panel.grid.major = element_line(color = "gray90"),
          legend.position = "top",
          legend.key.width = unit(1.5, "cm"),
          legend.direction = "vertical") +
    coord_fixed() +
    scale_linetype(name = "Term") +
    scale_color_viridis_d(name = "Term", option = "cividis") +
    guides(color = guide_legend(ncol = 1)) +
    labs(x = "Fixed exposure-outcome hazard ratio", 
         y = "Observed hazard ratio (95% CI)")
}

# Calculate correlations between confounders, between exposure and 
# confounders by true exposure-outcome hazard ratio
calculate_correlations <- function(df, excludevars = "truehr") {
  res <- df %>%
    mutate(
      cor.gleason.ptnm     = map_dbl(.x = data, 
                                     .f = ~cor.test(as.numeric(.$gleason),
                                                    as.numeric(.$ptnm_miss2))$estimate),
      cor.exposure.gleason = map_dbl(.x = data, 
                                     .f = ~cor.test(.$exposure, 
                                                    as.numeric(.$gleason))$estimate),
      cor.exposure.ptnm    = map_dbl(.x = data, 
                                     .f = ~cor.test(.$exposure, 
                                                    as.numeric(.$ptnm_miss2))$estimate)) %>%
    summarize_at(.vars = vars(starts_with("cor.")), 
                 .funs = list(median = median, 
                              ll     = ~quantile(., probs = 0.025), 
                              ul     = ~quantile(., probs = 0.975))) %>%
    pivot_longer(cols = -one_of(excludevars)) %>%
    separate(col = name, sep = "_", into = c("correlation", "statistic")) %>%
    pivot_wider(names_from = statistic, values_from = value)
  if(nrow(df) == 1)  # if not from simulations, but just on the one real dataset
    res <- res %>% select(-ll, -ul)
  res
}

# Plot those correlations
plot_correlations <- function(data) {
  data %>%
    ggplot(mapping = aes(x = truehr, y = median, 
                         ymin = ll, ymax = ul, group = correlation)) +
    geom_line(mapping = aes(color = correlation, linetype = correlation), size = 1) +
    geom_ribbon(alpha = 0.1) +
    scale_x_continuous(trans = "log", limits = c(0.33, 3),
                       breaks = c(0.33, 0.5, 1, 2, 3), expand = c(0, 0)) +
    theme(panel.grid.major = element_line(color = "gray90"),
          legend.position = "top",
          legend.key.width = unit(1.5, "cm"),
          legend.direction = "vertical") +
    scale_color_viridis_d(option = "cividis", name = "Term") +
    scale_linetype(name = "Term") +
    guides(color = guide_legend(ncol = 1),
           linetype = guide_legend(ncol = 1)) +
    labs(x = "Fixed exposure-outcome hazard ratio", 
         y = "Pearson correlation coefficient (95% CI)")
}

truecor <- dfexp2 %>%
  nest(data = everything()) %>%
  calculate_correlations()

get_truecor <- function(corr) {
  truecor %>%
    filter(correlation == corr) %>%
    pull(median) %>%
    as.numeric(.) %>%
    round(., digits = 2) %>%
    format(., nsmall = 2)
}
recode_corr <- c(
  "cor.gleason.ptnm"     = paste("Gleason--pTNM, true r =", 
                                 get_truecor("cor.gleason.ptnm")),
  "cor.exposure.gleason" = paste("Exposure--Gleason, true r =",
                                 get_truecor("cor.exposure.gleason")),
  "cor.exposure.ptnm"    = paste("Exposure--pTNM, true r =",
                                 get_truecor("cor.exposure.ptnm")))

part3 <- dfsim2 %>%
  group_by(truehr) %>%
  calculate_correlations() %>%
  mutate(correlation = recode(correlation, !!!recode_corr)) %>%
  plot_correlations()

part4 <- dfsim2 %>%
  plot_true_vs_obs_hrs(
    predictors = "exposure + as.numeric(gleason) + as.numeric(ptnm_miss2)",
    rename_list = c("exposure"               = "Exposure",
                    "as.numeric(gleason)"    = "Gleason score",
                    "as.numeric(ptnm_miss2)" = "pTNM stage"))

plot_grid(part1$plot, part2$plot, part3, part4, 
          ncol = 2, nrow = 2,
          align = "tlbr", axis = "hv", labels = LETTERS)
```

## Figure Supplement 2

The data correlation structure “confounding and imbalance.” Tumors with more extreme Gleason scores were set to be more extremely influenced by batch effects in terms of mean and variances.

```
dfexp2 %>% 
  rename(Gleason = gleason) %>%
  mutate(tma = factor(tma)) %>%
  stripplot(x = tma, y = exposureDbtc, color = Gleason) +
  labs(x = "Tissue microarray", y = "Biomarker [SDs]", color = "Gleason") +
  scale_color_viridis_d(option = "cividis") +
  scale_y_continuous(limits = c(min(c(dfexp2$exposure, 
                                      dfexp2$exposureBbtc, dfexp2$exposureCbtc)),
                                max(c(dfexp2$exposure, 
                                      dfexp2$exposureBbtc, dfexp2$exposureCbtc))),
                     breaks = seq(from = -4, to = 4, by = 1)) +
  theme(plot.title = element_text(size = 12),
        panel.grid.major.y = element_line(color = "gray90"))
```

## Figure Supplement 3

Plasmode simulation results for all scenarios. Observed hazard ratios after different approaches to batch effect correction (*y* axis) are compared to true (fixed) hazard ratios for the biomarker–outcome association (*x* axis; solid blue line: no correction for batch effects). Columns are different batch effects that were added; rows are different data correlation structures.

```
allhrs %>%
  plot_adj_hrs() +
  facet_grid(datalabel ~ batcheff) +
  theme(panel.spacing.x =  unit(0.6, "cm"),
        panel.spacing.y =  unit(0.4, "cm"),
        legend.key.width = unit(1.5, "cm"))
```

# Figure 6

**Consequences of batch effect mitigation on scientific inference.** **A**, Gleason score and biomarker levels (in standard deviations, per Gleason grade group). **B**, Biomarker levels and progression to lethal disease, with hazard ratios per one standard deviation increase in biomarker levels from univariable Cox regression models. In both panels, blue dots indicate estimates using uncorrected biomarker levels, yellow dots indicate batch effect-corrected levels, applying approach (6), quantile normalization. Lines are 95% confidence intervals. Biomarkers are ordered by decreasing intraclass correlation coefficient (ICC).

```
# Run linear model and return broomed list of coefficients
lmbroom <- function(data, formula, 
                    exponentiate = FALSE,  # exp(coef) when fitting a log-transformed outcome
                    digits       = 2) {    # digits to round estimate and CI limits to
  outcome <- if_else(typeof(formula) == "language", 
                     as.character(formula)[2], 
                     strsplit(as.character(formula), "~", fixed = TRUE)[[1]][1])
  data %>% 
    lm(formula = formula) %>%
    tidy(conf.int = TRUE, exponentiate = exponentiate) %>%
    select(term, estimate, conf.low, conf.high)
}

lmbroom_gleason <- function(marker, suffix = "") {
  lmbroom(data = comb,
          formula = as.formula(paste0(marker, suffix, "~ as.numeric(gleason)"))) %>% 
    select(-term) %>% 
    slice(2)
}

poss_lmbroom_gleason <- possibly(
  .f = lmbroom_gleason,
  otherwise = tibble(estimate = NA, conf.low = NA, conf.high = NA))

part_a <- markersfig2 %>%
  select(-scoring, -int, -cells, -area) %>%
  expand_grid(tibble(suffix = c("", "_adj6"))) %>%
  mutate(coefs = map2(.x = var_use, .y = suffix, .f = poss_lmbroom_gleason)) %>%
  unnest(cols = coefs) %>%
  mutate(adjust = factor(if_else(suffix == "", FALSE, TRUE),
                         levels = c(FALSE, TRUE),
                         labels = c("Uncorrected", "Quantile normalization"))) %>%
  mutate(ypos  = as.numeric(fct_rev(name)) + 
           if_else(adjust == "Uncorrected", 0.2, -0.2)) %>%
  ggplot(mapping = aes(x = estimate, xmin = conf.low, xmax = conf.high, 
                       y = ypos, color = adjust)) +
  geom_vline(xintercept = 0, linetype = "dashed", color = "black") +
  geom_errorbarh(size = 0.5, height = 0) +
  geom_point(size = 3) +
  scale_color_viridis_d(name = "Batch correction", option = "cividis", end = 0.8) +
  scale_x_continuous(limits = c(-0.3, 0.35), expand = c(0, 0.06), 
                     breaks = seq(from = -0.3, to = 0.3, by = 0.1),
                     labels = c("-0.3", "-0.2", "-0.1", "0", "0.1", "0.2", "0.3")) +
  scale_y_continuous(breaks = 1:nlevels(factor(markersfig2$name)),
                     labels = levels(fct_rev(factor(markersfig2$name))),
                     expand = c(0.03, 0.01)) +
  labs(x = "Difference in biomarker per Gleason grade group [SDs] (95% CI)", 
       y = "Increasing ICC") +
  theme(panel.grid.major.x = element_line(color = "gray85"),
        panel.grid.minor.x = element_line(color = "gray95"),
        legend.position = c(0.75, 0.25)) +
  geom_segment(x       = -0.463,
               xend    = -0.463, 
               yend    = nlevels(factor(markersfig2$name)) / 2 + 6, 
               y       = nlevels(factor(markersfig2$name)) / 2 - 6, 
               lineend = "butt", linejoin = "mitre", color = "black", 
               arrow = arrow(length = unit(0.3, "cm"))) +
  coord_cartesian(clip = "off")

# Obtaining Cox model coefficients
coxbroom <- function(data, 
                     exposure,            # exposure variable ... and all confounders
                     time   = "lethalfu", # time scale variable
                     event  = "eventlethal",   # outcome
                     round  = TRUE,       # round and format as a character?
                     digits = 2) {          # for rounding and formatting
  result <- data %>% 
    coxph(formula = as.formula(paste0("Surv(", time, ", ", event, ") ~ ", exposure))) 
  result <- result %>%
    tidy(conf.int = TRUE, exponentiate = TRUE) %>%
    select(term, estimate, conf.low, conf.high)
  if(round == TRUE)
    result <- result %>% mutate_at(.vars = vars(estimate, conf.low, conf.high), 
                                   .funs = ~format(round(., digits), nsmall = digits))
  result
}

coxextreme <- function(marker, suffix) {
  comb %>%
    rename(exposure = one_of(paste0(marker, suffix))) %>%
    mutate(quartiles = factor(as.numeric(factor(cut2(x = exposure, g = 4))))) %>%
    coxbroom(exposure = "quartiles", round = FALSE) %>%
    filter(term == "quartiles4") %>%
    select(-term)
}

pos_coxextreme <- possibly(.f = coxextreme, 
                            otherwise = tibble(estimate = NA, 
                                               conf.low = NA, conf.high = NA))
pos_coxbroom <- possibly(.f = coxbroom, 
                         otherwise = tibble(term = "", estimate = NA, 
                                            conf.low = NA, conf.high = NA))

part_b <- markersfig2 %>%
  select(-scoring, -int, -cells, -area) %>%
  expand_grid(tibble(suffix = c("", "_adj6"))) %>%
  mutate(
    coefs_linear = map2(.x = var_use, .y = suffix, 
                        .f = ~pos_coxbroom(data = comb, 
                                           exposure = paste0(.x, .y), 
                                           round = FALSE)),
    coefs_extr = map2(.x = var_use, .y = suffix, 
                      .f = pos_coxextreme)) %>%
    unnest(cols = coefs_linear) %>%
  mutate(adjust = factor(if_else(suffix == "", FALSE, TRUE),
                         levels = c(FALSE, TRUE),
                         labels = c("Uncorrected", "Quantile normalization"))) %>%
  mutate(ypos  = as.numeric(fct_rev(name)) + 
           if_else(adjust == "Uncorrected", 0.2, -0.2)) %>%
  ggplot(mapping = aes(x = estimate, xmin = conf.low, xmax = conf.high, 
                       y = ypos, color = adjust)) +
  geom_vline(xintercept = 1, linetype = "dashed", color = "black") +
  geom_errorbarh(size = 0.5, height = 0) +
  geom_point(size = 3) +
  scale_color_viridis_d(name = "Batch correction", option = "cividis", end = 0.8) +
  scale_x_continuous(trans = "log", 
                     limits = c(0.35, 3.3),
                     expand = c(0, 0),
                     breaks = c(0.33, 1/2, 1/1.25, 1, 1.25, 2, 3),
                     labels = c("0.33", "0.5", "0.8", "1", 
                                "1.25", "2", "3")) +
  scale_y_continuous(breaks = 1:nlevels(factor(markersfig2$name)),
                     labels = levels(fct_rev(factor(markersfig2$name))),
                     expand = c(0.03, 0.01)) +
  labs(x = "Hazard ratio per standard deviation of biomarker (95% CI)", 
       y = "Increasing ICC") +
  theme(panel.grid.major.x = element_line(color = "gray90"),
        legend.position = c(0.75, 0.25)) +
  geom_segment(x       = -1.361,
               xend    = -1.361, 
               yend    = nlevels(factor(markersfig2$name)) / 2 + 6, 
               y       = nlevels(factor(markersfig2$name)) / 2 - 6, 
               lineend = "butt", linejoin = "mitre", color = "black", 
               arrow = arrow(length = unit(0.3, "cm"))) +
  coord_cartesian(clip = "off")

plot_grid(part_a, part_b,
          ncol = 1, rel_heights = c(1, 1), labels = c("A", "B"))
```

# Figure 7

**Recommended workflow for anticipating and handling batch effects between tissue microarrays.** Following prevention approaches at the design phase of a project, all tissue microarray-based studies should explore the potential for batch effects once a biomarker has been measured. Addressing batch effects should only be skipped there is no indication for their presence. Batch effect-corrected biomarker levels can easily be generated by the batchtma R package.

# Supplementary File 1

## Table 1

Interaction terms to test for multiplicative effect modification, i.e. whether batch effects more strongly affect tumors with more extreme clinical/pathological characteristics. The table shows point estimates (differences in biomarker levels), 95% confidence interval bounds, p-values, and false-discovery rates (FDR) for interaction terms between the within-batch normalized biomarker level and the potential effect modifier in linear models that have absolute biomarker levels in standard deviation units per biomarker as the outcome and also include main effects for the biomarker and the effect modifier (terms not shown).

```
df <- comb %>%
  group_by(tma) %>%
  mutate_at(.vars = vars(all_of(markers$var_use)),
            .funs = list(
              batchz       = ~as.numeric(scale(.)), 
              batchzabs    = ~abs(as.numeric(scale(.))), 
              batchmean    = ~mean(., na.rm = TRUE),
              batchmeanabs = ~abs(mean(., na.rm = TRUE)))) %>%
  ungroup()

markers %>% 
  select(name, var_use) %>%
  expand_grid(confounder = c("as.numeric(gleason)", 
                             "as.numeric(ptnm_miss2)", 
                             "dxyear")) %>%
  mutate(model = map2(
    .x = var_use, .y = confounder,
    .f = ~{ 
      df %>% 
        lme4::lmer(formula = as.formula(paste0(.x, "_batchz ~ ", .y, " + ", 
                                               .x, "_batchmean + ", 
                                               .y, "*", .x, 
                                               "_batchmean + (1 | tma)"))) %>%
        broom.mixed::tidy(conf.int = TRUE) %>%
        slice(4) })) %>%
  unnest(cols = model) %>%
  mutate(pvalue = 2 * pnorm(abs(statistic), lower.tail = FALSE),
         fdr = p.adjust(pvalue, method = "BH")) %>%
  arrange(pvalue) %>%
  mutate(p_real = pvalue) %>%
  mutate_at(.vars = vars(estimate, conf.low, conf.high),
            .funs = ~trimws(format(round(., digits = 2), nsmall = 2))) %>%
  mutate_at(.vars = vars(pvalue, fdr),
            .funs = ~format(round(., digits = 3), nsmall = 3)) %>%
  mutate(`Estimate (95% CI)` = paste0(estimate, " (", conf.low, " to ", conf.high, ")"),
         confounder = recode(confounder, 
                             "dxyear"                 = "Calendar year",
                             "as.numeric(ptnm_miss2)" = "pTNM group",
                             "as.numeric(gleason)"    = "Gleason score")) %>%
  mutate(p = if_else(condition = pvalue == "0.000", 
                     true = formatC(p_real, format = "e", digits = 1), 
                     false = pvalue)) %>%
  select(Biomarker = name, `Effect modifier` = confounder,
         `Estimate (95% CI)`, p, FDR = fdr) %>%
  mygt()
```

| Biomarker | Effect modifier | Estimate (95% CI) | p | FDR |
| --- | --- | --- | --- | --- |
| Ki-67 | pTNM group | 0.41 (0.20 to 0.63) | 2.0e-04 | 0.012 |
| Beta-catenin | pTNM group | 0.36 (0.00 to 0.71) | 0.047 | 0.629 |
| TUNEL | Calendar year | 0.08 (0.00 to 0.16) | 0.050 | 0.629 |
| SMAD4 | Calendar year | -0.07 (-0.14 to 0.00) | 0.055 | 0.629 |
| Androgen R | pTNM group | 0.11 (-0.01 to 0.23) | 0.066 | 0.629 |
| ER-beta | Calendar year | -0.02 (-0.05 to 0.00) | 0.074 | 0.629 |
| ER-beta | pTNM group | -0.08 (-0.18 to 0.01) | 0.089 | 0.629 |
| Beta-catenin | Calendar year | -0.08 (-0.17 to 0.02) | 0.100 | 0.629 |
| IGF1 R | Gleason score | 0.14 (-0.03 to 0.30) | 0.105 | 0.629 |
| Calcium SR | Calendar year | 0.02 (-0.01 to 0.04) | 0.120 | 0.629 |
| Androgen R | Gleason score | 0.08 (-0.02 to 0.17) | 0.120 | 0.629 |
| Vitamin D R | Calendar year | -0.03 (-0.07 to 0.01) | 0.140 | 0.629 |
| Osteopontin | Calendar year | 0.05 (-0.02 to 0.11) | 0.145 | 0.629 |
| Ki-67 | Calendar year | -0.04 (-0.10 to 0.02) | 0.147 | 0.629 |
| MYC | Calendar year | -0.05 (-0.11 to 0.02) | 0.175 | 0.702 |
| MYC | pTNM group | -0.16 (-0.41 to 0.09) | 0.209 | 0.767 |
| IGF1 R | Calendar year | 0.03 (-0.02 to 0.08) | 0.217 | 0.767 |
| PSMA | Gleason score | -0.25 (-0.66 to 0.17) | 0.247 | 0.797 |
| Insulin R | Calendar year | -0.04 (-0.12 to 0.03) | 0.253 | 0.797 |
| FASN | Calendar year | -0.02 (-0.06 to 0.02) | 0.290 | 0.824 |
| Osteopontin | Gleason score | -0.11 (-0.31 to 0.10) | 0.302 | 0.824 |
| Adiponectin R2 | Gleason score | -0.07 (-0.19 to 0.06) | 0.321 | 0.824 |
| ER-alpha | Gleason score | 0.04 (-0.04 to 0.12) | 0.323 | 0.824 |
| PSMA | Calendar year | 0.07 (-0.08 to 0.23) | 0.352 | 0.824 |
| Adiponectin R2 | Calendar year | -0.02 (-0.06 to 0.02) | 0.352 | 0.824 |
| PTEN-IF | pTNM group | -0.08 (-0.26 to 0.09) | 0.357 | 0.824 |
| Calcium SR | pTNM group | -0.04 (-0.12 to 0.05) | 0.393 | 0.864 |
| PSMA | pTNM group | -0.23 (-0.76 to 0.31) | 0.403 | 0.864 |
| Stathmin-IF | pTNM group | -0.04 (-0.13 to 0.06) | 0.450 | 0.930 |
| FASN | pTNM group | 0.05 (-0.10 to 0.21) | 0.486 | 0.931 |
| Stathmin-IF | Calendar year | 0.01 (-0.01 to 0.03) | 0.504 | 0.931 |
| SMAD4 | pTNM group | 0.09 (-0.18 to 0.36) | 0.521 | 0.931 |
| PTEN-IF | Calendar year | -0.01 (-0.05 to 0.03) | 0.545 | 0.931 |
| Osteopontin | pTNM group | -0.07 (-0.28 to 0.15) | 0.554 | 0.931 |
| Vitamin D R | Gleason score | -0.03 (-0.15 to 0.08) | 0.585 | 0.931 |
| pS6-IF | Gleason score | -0.04 (-0.20 to 0.11) | 0.605 | 0.931 |
| TUNEL | pTNM group | -0.09 (-0.43 to 0.26) | 0.618 | 0.931 |
| pS6-IF | pTNM group | 0.05 (-0.15 to 0.24) | 0.645 | 0.931 |
| MYC | Gleason score | 0.05 (-0.16 to 0.25) | 0.652 | 0.931 |
| FASN | Gleason score | 0.03 (-0.10 to 0.15) | 0.665 | 0.931 |
| SMAD4 | Gleason score | -0.05 (-0.25 to 0.16) | 0.667 | 0.931 |
| IGF1 R | pTNM group | -0.04 (-0.23 to 0.15) | 0.673 | 0.931 |
| Androgen R | Calendar year | 0.01 (-0.02 to 0.04) | 0.676 | 0.931 |
| pS6-IF | Calendar year | -0.01 (-0.05 to 0.03) | 0.729 | 0.931 |
| Cyclin D1 | Calendar year | 0.02 (-0.08 to 0.11) | 0.736 | 0.931 |
| TUNEL | Gleason score | -0.04 (-0.32 to 0.23) | 0.750 | 0.931 |
| Cyclin D1 | Gleason score | 0.05 (-0.28 to 0.37) | 0.775 | 0.931 |
| ER-alpha | pTNM group | -0.01 (-0.11 to 0.08) | 0.777 | 0.931 |
| Insulin R | Gleason score | 0.03 (-0.20 to 0.27) | 0.782 | 0.931 |
| Calcium SR | Gleason score | -0.01 (-0.08 to 0.07) | 0.808 | 0.931 |
| Stathmin-IF | Gleason score | -0.01 (-0.09 to 0.07) | 0.809 | 0.931 |
| ER-beta | Gleason score | 0.01 (-0.07 to 0.09) | 0.828 | 0.931 |
| ER-alpha | Calendar year | 0.00 (-0.02 to 0.02) | 0.836 | 0.931 |
| Adiponectin R2 | pTNM group | 0.02 (-0.14 to 0.17) | 0.838 | 0.931 |
| Insulin R | pTNM group | -0.02 (-0.30 to 0.26) | 0.888 | 0.965 |
| Vitamin D R | pTNM group | -0.01 (-0.15 to 0.14) | 0.929 | 0.965 |
| PTEN-IF | Gleason score | 0.01 (-0.15 to 0.16) | 0.930 | 0.965 |
| Ki-67 | Gleason score | 0.01 (-0.19 to 0.20) | 0.933 | 0.965 |
| Beta-catenin | Gleason score | -0.01 (-0.28 to 0.26) | 0.951 | 0.967 |
| Cyclin D1 | pTNM group | 0.01 (-0.36 to 0.38) | 0.973 | 0.973 |

## Table 2

Intraclass correlation coefficient (ICC) for between-batch variance for uncorrected biomarker levels (“1 Raw”) and biomarker levels after applying different correction methods.

```
wrap_icc_oneway <- function(marker) {
  res <- icc_oneway(marker, data = comb) %>%
    as.numeric() %>%
    round(., digits = 2) %>%
    format(., nsmall = 2)
  paste0(res[1], " (", res[2], "-", res[3], ")")
}

# catch failing ICC calculation (due to missing adjusted IFG1R values)
pos_wrap_icc_oneway <- possibly(.f = wrap_icc_oneway, otherwise = "N/A")

icc_after <- markers %>%
  select(var_use, name) %>%
  mutate(`1 Uncorrected`            = map_chr(.x = var_use,                  
                                              .f = pos_wrap_icc_oneway),
         `2 Simple mean`            = map_chr(.x = paste0(var_use, "_adj2"), 
                                              .f = pos_wrap_icc_oneway),
         `3 Standardized mean`      = map_chr(.x = paste0(var_use, "_adj3"), 
                                              .f = pos_wrap_icc_oneway),
         `4 IP-weighted mean`       = map_chr(.x = paste0(var_use, "_adj4"), 
                                              .f = pos_wrap_icc_oneway),
         `5 Quantile regression`    = map_chr(.x = paste0(var_use, "_adj5"), 
                                              .f = pos_wrap_icc_oneway),
         `6 Quantile normalization` = map_chr(.x = paste0(var_use, "_adj6"), 
                                              .f = pos_wrap_icc_oneway),
         `7 ComBat`                 = map_chr(.x = paste0(var_use, "_adj7"), 
                                              .f = pos_wrap_icc_oneway)) %>%
  select(-var_use) %>%
  rename(Biomarker = name)
```

```
icc_after %>% mygt()
```

| Biomarker | 1 Uncorrected | 2 Simple mean | 3 Standardized mean | 4 IP-weighted mean | 5 Quantile regression | 6 Quantile normalization | 7 ComBat |
| --- | --- | --- | --- | --- | --- | --- | --- |
| Adiponectin R2 | 0.20 (0.05-0.36) | 0.00 (0.00-0.00) | 0.00 (0.00-0.01) | 0.00 (0.00-0.01) | 0.01 (0.00-0.03) | 0.00 (0.00-0.01) | 0.00 (0.00-0.01) |
| Androgen R | 0.31 (0.09-0.50) | 0.00 (0.00-0.00) | 0.00 (0.00-0.01) | 0.00 (0.00-0.01) | 0.00 (0.00-0.01) | 0.00 (0.00-0.01) | 0.00 (0.00-0.01) |
| Beta-catenin | 0.03 (0.00-0.08) | 0.00 (0.00-0.00) | 0.00 (0.00-0.02) | 0.00 (0.00-0.01) | 0.02 (0.00-0.06) | 0.00 (0.00-0.01) | 0.00 (0.00-0.02) |
| Calcium SR | 0.36 (0.16-0.52) | 0.00 (0.00-0.00) | 0.00 (0.00-0.01) | 0.00 (0.00-0.01) | 0.01 (0.00-0.02) | 0.00 (0.00-0.01) | 0.00 (0.00-0.01) |
| Cyclin D1 | 0.02 (0.00-0.07) | 0.00 (0.00-0.00) | 0.00 (0.00-0.01) | 0.00 (0.00-0.01) | 0.00 (0.00-0.01) | 0.00 (0.00-0.01) | 0.00 (0.00-0.01) |
| ER-alpha | 0.41 (0.19-0.57) | 0.00 (0.00-0.00) | 0.00 (0.00-0.01) | 0.00 (0.00-0.00) | 0.00 (0.00-0.01) | 0.00 (0.00-0.01) | 0.00 (0.00-0.01) |
| ER-beta | 0.37 (0.16-0.53) | 0.00 (0.00-0.00) | 0.00 (0.00-0.01) | 0.00 (0.00-0.01) | 0.00 (0.00-0.01) | 0.00 (0.00-0.01) | 0.00 (0.00-0.01) |
| FASN | 0.18 (0.05-0.33) | 0.00 (0.00-0.00) | 0.00 (0.00-0.01) | 0.00 (0.00-0.01) | 0.00 (0.00-0.01) | 0.00 (0.00-0.01) | 0.00 (0.00-0.01) |
| IGF1 R | 0.13 (0.03-0.26) | 0.00 (0.00-0.00) | 0.00 (0.00-0.01) | 0.00 (0.00-0.01) | 0.03 (0.00-0.08) | 0.00 (0.00-0.01) | 0.00 (0.00-0.01) |
| Insulin R | 0.05 (0.00-0.13) | 0.00 (0.00-0.00) | 0.00 (0.00-0.01) | 0.00 (0.00-0.01) | 0.00 (0.00-0.01) | 0.00 (0.00-0.01) | 0.00 (0.00-0.01) |
| Ki-67 | 0.08 (0.01-0.16) | 0.00 (0.00-0.00) | 0.01 (0.00-0.03) | 0.02 (0.00-0.06) | 0.01 (0.00-0.04) | 0.00 (0.00-0.01) | 0.01 (0.00-0.03) |
| MYC | 0.09 (0.01-0.19) | 0.00 (0.00-0.00) | 0.00 (0.00-0.01) | 0.00 (0.00-0.01) | 0.00 (0.00-0.01) | 0.00 (0.00-0.01) | 0.00 (0.00-0.01) |
| Osteopontin | 0.10 (0.02-0.20) | 0.00 (0.00-0.00) | 0.00 (0.00-0.01) | 0.00 (0.00-0.01) | 0.00 (0.00-0.01) | 0.00 (0.00-0.01) | 0.00 (0.00-0.01) |
| PSMA | 0.01 (0.00-0.03) | 0.00 (0.00-0.00) | 0.00 (0.00-0.01) | 0.00 (0.00-0.01) | 0.00 (0.00-0.01) | 0.00 (0.00-0.01) | 0.00 (0.00-0.01) |
| PTEN-IF | 0.13 (0.04-0.23) | 0.00 (0.00-0.00) | 0.00 (0.00-0.01) | 0.00 (0.00-0.01) | 0.00 (0.00-0.01) | 0.00 (0.00-0.01) | 0.00 (0.00-0.01) |
| SMAD4 | 0.07 (0.01-0.15) | 0.00 (0.00-0.00) | 0.00 (0.00-0.02) | 0.00 (0.00-0.02) | 0.01 (0.00-0.03) | 0.00 (0.00-0.02) | 0.00 (0.00-0.02) |
| Stathmin-IF | 0.48 (0.23-0.63) | 0.00 (0.00-0.00) | 0.00 (0.00-0.01) | 0.00 (0.00-0.01) | 0.01 (0.00-0.03) | 0.00 (0.00-0.01) | 0.00 (0.00-0.01) |
| pS6-IF | 0.08 (0.02-0.15) | 0.00 (0.00-0.00) | 0.00 (0.00-0.01) | 0.00 (0.00-0.01) | 0.00 (0.00-0.01) | 0.00 (0.00-0.01) | 0.00 (0.00-0.01) |
| TUNEL | 0.04 (0.00-0.10) | 0.00 (0.00-0.00) | 0.00 (0.00-0.01) | 0.00 (0.00-0.01) | 0.10 (0.02-0.20) | 0.00 (0.00-0.01) | 0.00 (0.00-0.01) |
| Vitamin D R | 0.24 (0.06-0.41) | 0.00 (0.00-0.00) | 0.00 (0.00-0.01) | 0.00 (0.00-0.01) | 0.00 (0.00-0.01) | 0.00 (0.00-0.01) | 0.00 (0.00-0.01) |

## Table 3

Results from plasmode simulation according to type of induced batch effect, using the data correlation structure “Some confounding.” For three fixed (“true”) hazard ratios for the biomarker–outcome association (1⁄3, 1, and 3), the observed hazard ratios after batch correction (with 95% confidence intervals) are shown.

```
hrsum <- function(data) {
  data %>%
    summarize_at(
      .vars = vars(contains("_adj")),
      .funs = ~paste0(format(round(exp(log(mean(., na.rm = TRUE))), 
                                   digits = 2), nsmall = 2),
                      " (",
                      format(round(exp(log(mean(., na.rm = TRUE)) - 
                                             qnorm(0.975) * sd(log(.), na.rm = TRUE)),
                                   digits = 2), nsmall = 2), 
                      "-",
                      format(round(exp(log(mean(., na.rm = TRUE)) + 
                                             qnorm(0.975) * sd(log(.), na.rm = TRUE)),
                                   digits = 2), nsmall = 2),
                      ")")) %>%
    rename_at(.vars = vars(contains("hr_adj")),
              .funs = ~model_labels[as.numeric(str_remove(string = ., 
                                                          pattern = "hr_adj"))]) %>%
    ungroup() %>%
    mutate(truehr = factor(truehr, levels = c(1/3, 1, 3),
                           labels = c("1/3", "1", "3"))) %>%
    rename_all(.funs = recode,  # rename variables but do not fail if variable does not exist
               truehr = "True HR", 
               batcheff = "Batch effect", 
               datalabel = "Correlation structure") %>%
    mygt()
}

allhrs %>%
  filter(datalabel == "Some confounding") %>%
  filter(truehr == 1/3 | truehr > 2.9 | (truehr > 0.999 & truehr <= 1)) %>%
  group_by(truehr, batcheff) %>%
  hrsum()
```

| True HR | Batch effect | 1 Uncorrected | 2 Simple mean | 3 Standardized mean | 4 IP-weighted mean | 5 Quantile regression | 6 Quantile normalization | 8 IV-pooled | 9 Strata |
| --- | --- | --- | --- | --- | --- | --- | --- | --- | --- |
| 1/3 | No batch effects | 0.33 (0.27-0.41) | 0.33 (0.27-0.41) | 0.33 (0.27-0.41) | 0.33 (0.27-0.41) | 0.28 (0.22-0.37) | 0.34 (0.27-0.42) | 0.29 (0.13-0.64) | 0.32 (0.26-0.41) |
| 1/3 | Mean only | 0.46 (0.39-0.55) | 0.33 (0.27-0.41) | 0.33 (0.27-0.41) | 0.33 (0.27-0.41) | 0.34 (0.27-0.42) | 0.34 (0.27-0.42) | 0.29 (0.13-0.64) | 0.32 (0.26-0.41) |
| 1/3 | Mean and variance | 0.44 (0.36-0.54) | 0.37 (0.30-0.45) | 0.37 (0.30-0.45) | 0.37 (0.30-0.45) | 0.34 (0.28-0.43) | 0.32 (0.26-0.41) | 0.32 (0.16-0.64) | 0.34 (0.27-0.44) |
| 1/3 | Effect modification | 0.49 (0.41-0.60) | 0.41 (0.34-0.50) | 0.42 (0.34-0.50) | 0.42 (0.35-0.51) | 0.38 (0.31-0.47) | 0.38 (0.31-0.47) | 0.36 (0.26-0.51) | 0.40 (0.32-0.49) |
| 1 | No batch effects | 0.99 (0.81-1.21) | 0.99 (0.81-1.21) | 0.99 (0.81-1.21) | 0.99 (0.81-1.21) | 0.99 (0.78-1.25) | 0.99 (0.81-1.22) | 0.98 (0.72-1.34) | 0.99 (0.80-1.22) |
| 1 | Mean only | 0.99 (0.85-1.17) | 0.99 (0.81-1.21) | 0.99 (0.81-1.21) | 0.99 (0.81-1.21) | 0.99 (0.81-1.21) | 0.99 (0.81-1.22) | 0.98 (0.72-1.34) | 0.99 (0.80-1.22) |
| 1 | Mean and variance | 0.99 (0.85-1.16) | 0.99 (0.82-1.20) | 0.99 (0.82-1.21) | 0.99 (0.82-1.21) | 0.99 (0.81-1.21) | 0.99 (0.80-1.23) | 0.99 (0.73-1.32) | 0.99 (0.81-1.22) |
| 1 | Effect modification | 1.00 (0.88-1.13) | 0.99 (0.85-1.16) | 0.99 (0.85-1.16) | 0.99 (0.85-1.16) | 0.99 (0.84-1.17) | 1.00 (0.84-1.18) | 1.00 (0.76-1.31) | 0.99 (0.84-1.17) |
| 3 | No batch effects | 3.09 (2.54-3.76) | 3.07 (2.53-3.74) | 3.08 (2.53-3.76) | 3.07 (2.52-3.74) | 3.70 (2.95-4.65) | 3.03 (2.48-3.69) | 3.56 (2.49-5.09) | 3.15 (2.55-3.90) |
| 3 | Mean only | 2.17 (1.86-2.53) | 3.07 (2.53-3.74) | 3.08 (2.53-3.76) | 3.07 (2.52-3.74) | 3.02 (2.49-3.66) | 3.03 (2.48-3.69) | 3.56 (2.49-5.09) | 3.15 (2.55-3.90) |
| 3 | Mean and variance | 1.90 (1.66-2.16) | 2.67 (2.25-3.17) | 2.70 (2.26-3.21) | 2.70 (2.26-3.22) | 2.97 (2.46-3.58) | 3.11 (2.54-3.81) | 3.33 (2.29-4.84) | 3.01 (2.41-3.76) |
| 3 | Effect modification | 1.66 (1.49-1.86) | 2.10 (1.82-2.42) | 2.12 (1.84-2.46) | 2.13 (1.84-2.47) | 2.39 (2.04-2.80) | 2.43 (2.06-2.87) | 3.13 (1.81-5.43) | 2.48 (2.05-2.99) |

## Table 4

Results from plasmode simulation according to data correlation structure, using the batch effect “mean and variance.” For three fixed (“true”) hazard ratios for the biomarker–outcome association (1⁄3, 1, and 3), the observed hazard ratios after batch correction (with 95% confidence intervals) are shown.

```
allhrs %>%
  filter(batcheff == "Mean and variance") %>%
  filter(truehr == 1/3 | truehr > 2.9 | (truehr > 0.999 & truehr <= 1)) %>%
  group_by(truehr, datalabel) %>%
  hrsum()
```

| True HR | Correlation structure | 1 Uncorrected | 2 Simple mean | 3 Standardized mean | 4 IP-weighted mean | 5 Quantile regression | 6 Quantile normalization | 8 IV-pooled | 9 Strata |
| --- | --- | --- | --- | --- | --- | --- | --- | --- | --- |
| 1/3 | No confounding | 0.43 (0.35-0.53) | 0.37 (0.31-0.46) | 0.37 (0.31-0.46) | 0.38 (0.31-0.46) | 0.40 (0.34-0.49) | 0.32 (0.26-0.40) | 0.31 (0.19-0.50) | 0.34 (0.27-0.43) |
| 1/3 | Some confounding | 0.44 (0.36-0.54) | 0.37 (0.30-0.45) | 0.37 (0.30-0.45) | 0.37 (0.30-0.45) | 0.34 (0.28-0.43) | 0.32 (0.26-0.41) | 0.32 (0.16-0.64) | 0.34 (0.27-0.44) |
| 1/3 | Moderate confounding | 0.45 (0.35-0.56) | 0.37 (0.30-0.46) | 0.37 (0.30-0.46) | 0.37 (0.30-0.46) | 0.30 (0.23-0.39) | 0.33 (0.26-0.42) | 0.32 (0.11-0.91) | 0.35 (0.27-0.45) |
| 1/3 | Confounding+imbalance | 0.44 (0.35-0.55) | 0.38 (0.31-0.47) | 0.37 (0.30-0.46) | 0.39 (0.31-0.48) | 0.30 (0.23-0.39) | 0.33 (0.26-0.41) | 0.32 (0.13-0.76) | 0.34 (0.26-0.45) |
| 1 | No confounding | 1.01 (0.85-1.19) | 1.00 (0.82-1.22) | 1.00 (0.82-1.22) | 1.00 (0.82-1.22) | 1.00 (0.85-1.19) | 1.01 (0.82-1.24) | 1.01 (0.76-1.34) | 1.00 (0.81-1.24) |
| 1 | Some confounding | 0.99 (0.85-1.16) | 0.99 (0.82-1.20) | 0.99 (0.82-1.21) | 0.99 (0.82-1.21) | 0.99 (0.81-1.21) | 0.99 (0.80-1.23) | 0.99 (0.73-1.32) | 0.99 (0.81-1.22) |
| 1 | Moderate confounding | 1.01 (0.87-1.18) | 1.01 (0.84-1.23) | 1.01 (0.84-1.23) | 1.01 (0.84-1.23) | 1.02 (0.82-1.26) | 1.01 (0.83-1.24) | 1.02 (0.75-1.38) | 1.01 (0.82-1.25) |
| 1 | Confounding+imbalance | 1.00 (0.85-1.17) | 1.01 (0.84-1.21) | 1.01 (0.83-1.21) | 1.01 (0.84-1.21) | 1.01 (0.82-1.24) | 1.01 (0.83-1.22) | 1.00 (0.68-1.47) | 1.01 (0.83-1.22) |
| 3 | No confounding | 1.95 (1.68-2.26) | 2.75 (2.29-3.31) | 2.75 (2.29-3.31) | 2.75 (2.29-3.31) | 2.55 (2.15-3.01) | 3.11 (2.53-3.82) | 3.38 (2.25-5.06) | 2.96 (2.41-3.64) |
| 3 | Some confounding | 1.90 (1.66-2.16) | 2.67 (2.25-3.17) | 2.70 (2.26-3.21) | 2.70 (2.26-3.22) | 2.97 (2.46-3.58) | 3.11 (2.54-3.81) | 3.33 (2.29-4.84) | 3.01 (2.41-3.76) |
| 3 | Moderate confounding | 1.82 (1.61-2.07) | 2.54 (2.13-3.03) | 2.60 (2.18-3.11) | 2.61 (2.18-3.12) | 3.36 (2.72-4.15) | 3.04 (2.50-3.69) | 10.78 (4.26-27.28) | 3.04 (2.42-3.82) |
| 3 | Confounding+imbalance | 1.86 (1.65-2.11) | 2.65 (2.22-3.15) | 2.63 (2.21-3.13) | 2.71 (2.27-3.24) | 3.31 (2.68-4.07) | 3.01 (2.46-3.67) | 3.40 (2.17-5.31) | 2.99 (2.40-3.72) |

## Table 5

Gleason grade—biomarker associations according to batch effect correction method. Point estimates from unadjusted linear regression models for biomarker values with Gleason score categories per 1 “grade group” increase as the predictor are shown (with 95% confidence intervals). For \(log\_e\)-transformed markers like Ki-67, estimates are differences on the loge scale.

```
pos_lmbroom <- possibly(
  .f = lmbroom, 
  otherwise = tibble(term      = c(" ", " "), 
                     estimate  = c(NA_real_, NA_real_), 
                     conf.low  = c(NA_real_, NA_real_), 
                     conf.high = c(NA_real_, NA_real_)))

markers %>%
  select(var_use, Marker = name) %>%
  mutate(res = map(.x = var_use, .f = ~{
    lmbroom(data = comb, formula = as.formula(paste0(.x, " ~ as.numeric(gleason)"))) %>%
      rename(adj1 = estimate, ll1 = conf.low, ul1 = conf.high) %>%
      left_join(lmbroom(data = comb, 
                        formula = as.formula(paste0(.x, "_adj2 ~ as.numeric(gleason)"))) %>%
                  rename(adj2 = estimate, ll2 = conf.low, ul2 = conf.high),
                by = "term") %>%
      left_join(lmbroom(data = comb, 
                        formula = as.formula(paste0(.x, "_adj3 ~ as.numeric(gleason)"))) %>%
                  rename(adj3 = estimate, ll3 = conf.low, ul3 = conf.high),
                by = "term") %>%
      left_join(pos_lmbroom(data = comb, 
                            formula = as.formula(paste0(.x, 
                                                        "_adj4 ~ as.numeric(gleason)"))) %>%
                  rename(adj4 = estimate, ll4 = conf.low, ul4 = conf.high),
                by = "term") %>%
      left_join(pos_lmbroom(data = comb, 
                            formula = as.formula(paste0(.x, 
                                                        "_adj5 ~ as.numeric(gleason)"))) %>%
                  rename(adj5 = estimate, ll5 = conf.low, ul5 = conf.high),
                by = "term") %>%
      left_join(lmbroom(data = comb, 
                        formula = as.formula(paste0(.x, "_adj6 ~ as.numeric(gleason)"))) %>%
                  rename(adj6 = estimate, ll6 = conf.low, ul6 = conf.high),
                by = "term") %>%
      left_join(pos_lmbroom(data = comb, 
                            formula = as.formula(paste0(.x, 
                                                        "_adj7 ~ as.numeric(gleason)"))) %>%
                  rename(adj7 = estimate, ll7 = conf.low, ul7 = conf.high),
                by = "term") %>%
      slice(-1) %>% select(-term) })) %>%
  unnest(cols = res) %>%
  mutate_at(.vars = vars(adj1:ul7), 
            .funs = ~trimws(format(round(., digits = 2), nsmall = 2))) %>%
  mutate(`1 Uncorrected`            = paste0(adj1, " (", ll1, " to ", ul1, ")"),
         `2 Simple mean`            = paste0(adj2, " (", ll2, " to ", ul2, ")"),
         `3 Standardized mean`      = paste0(adj3, " (", ll3, " to ", ul3, ")"),
         `4 IP-weighted mean`       = paste0(adj4, " (", ll4, " to ", ul4, ")"),
         `5 Quantile regression`    = paste0(adj5, " (", ll5, " to ", ul5, ")"),
         `6 Quantile normalization` = paste0(adj6, " (", ll6, " to ", ul6, ")"),
         `7 ComBat`                 = paste0(adj7, " (", ll7, " to ", ul7, ")")) %>%
  select(-var_use, -adj1:-ul7) %>%
  mygt()
```

| Marker | 1 Uncorrected | 2 Simple mean | 3 Standardized mean | 4 IP-weighted mean | 5 Quantile regression | 6 Quantile normalization | 7 ComBat |
| --- | --- | --- | --- | --- | --- | --- | --- |
| Adiponectin R2 | 0.07 (0.02 to 0.13) | 0.04 (-0.01 to 0.09) | 0.04 (0.00 to 0.09) | 0.04 (-0.01 to 0.09) | 0.04 (0.00 to 0.09) | 0.04 (-0.01 to 0.08) | 0.05 (0.00 to 0.09) |
| Androgen R | 0.03 (-0.03 to 0.08) | 0.01 (-0.04 to 0.05) | 0.01 (-0.04 to 0.05) | 0.01 (-0.04 to 0.05) | 0.00 (-0.05 to 0.05) | 0.01 (-0.03 to 0.05) | 0.01 (-0.04 to 0.05) |
| Beta-catenin | -0.17 (-0.23 to -0.12) | -0.17 (-0.23 to -0.12) | -0.18 (-0.24 to -0.13) | -0.17 (-0.23 to -0.12) | -0.14 (-0.18 to -0.10) | -0.16 (-0.21 to -0.11) | -0.18 (-0.23 to -0.13) |
| Calcium SR | 0.00 (-0.04 to 0.05) | 0.01 (-0.02 to 0.04) | 0.01 (-0.02 to 0.04) | 0.01 (-0.02 to 0.04) | 0.02 (-0.02 to 0.06) | 0.01 (-0.02 to 0.04) | 0.01 (-0.02 to 0.05) |
| Cyclin D1 | 0.06 (0.00 to 0.12) | 0.07 (0.01 to 0.13) | 0.07 (0.01 to 0.13) | 0.07 (0.01 to 0.13) | 0.07 (0.02 to 0.13) | 0.07 (0.01 to 0.12) | 0.07 (0.01 to 0.13) |
| ER-alpha | -0.04 (-0.09 to 0.01) | 0.02 (-0.02 to 0.06) | 0.02 (-0.02 to 0.06) | 0.02 (-0.02 to 0.06) | 0.02 (-0.03 to 0.07) | 0.02 (-0.02 to 0.05) | 0.02 (-0.02 to 0.05) |
| ER-beta | -0.02 (-0.07 to 0.03) | -0.01 (-0.05 to 0.02) | -0.02 (-0.06 to 0.02) | -0.01 (-0.05 to 0.02) | -0.02 (-0.06 to 0.02) | -0.01 (-0.05 to 0.03) | -0.02 (-0.06 to 0.02) |
| FASN | -0.05 (-0.11 to 0.00) | -0.07 (-0.12 to -0.03) | -0.08 (-0.12 to -0.03) | -0.08 (-0.13 to -0.03) | -0.08 (-0.13 to -0.03) | -0.07 (-0.12 to -0.02) | -0.07 (-0.12 to -0.03) |
| IGF1 R | -0.01 (-0.07 to 0.05) | -0.03 (-0.09 to 0.02) | -0.03 (-0.09 to 0.02) | -0.04 (-0.09 to 0.02) | -0.03 (-0.09 to 0.02) | -0.03 (-0.08 to 0.02) | -0.03 (-0.09 to 0.02) |
| Insulin R | 0.04 (-0.02 to 0.10) | 0.02 (-0.04 to 0.08) | 0.02 (-0.04 to 0.08) | 0.02 (-0.04 to 0.08) | 0.02 (-0.03 to 0.08) | 0.02 (-0.04 to 0.08) | 0.02 (-0.04 to 0.08) |
| Ki-67 | 0.10 (0.05 to 0.15) | 0.10 (0.05 to 0.15) | 0.09 (0.04 to 0.15) | 0.09 (0.04 to 0.15) | 0.11 (0.05 to 0.16) | 0.10 (0.05 to 0.16) | 0.09 (0.04 to 0.15) |
| MYC | 0.00 (-0.06 to 0.06) | -0.04 (-0.10 to 0.02) | -0.04 (-0.10 to 0.02) | -0.04 (-0.10 to 0.02) | -0.03 (-0.09 to 0.02) | -0.03 (-0.09 to 0.02) | -0.04 (-0.10 to 0.02) |
| Osteopontin | 0.18 (0.12 to 0.24) | 0.15 (0.10 to 0.21) | 0.16 (0.10 to 0.22) | 0.16 (0.10 to 0.21) | 0.13 (0.08 to 0.19) | 0.14 (0.08 to 0.19) | 0.16 (0.10 to 0.21) |
| PSMA | 0.23 (0.18 to 0.28) | 0.23 (0.17 to 0.28) | 0.23 (0.18 to 0.29) | 0.23 (0.18 to 0.28) | 0.18 (0.14 to 0.22) | 0.23 (0.18 to 0.28) | 0.24 (0.18 to 0.29) |
| PTEN-IF | -0.09 (-0.14 to -0.04) | -0.08 (-0.13 to -0.03) | -0.08 (-0.12 to -0.03) | -0.08 (-0.13 to -0.03) | -0.07 (-0.12 to -0.03) | -0.08 (-0.12 to -0.04) | -0.07 (-0.12 to -0.02) |
| SMAD4 | -0.07 (-0.12 to -0.01) | -0.07 (-0.12 to -0.01) | -0.07 (-0.13 to -0.02) | -0.07 (-0.13 to -0.02) | -0.08 (-0.13 to -0.02) | -0.06 (-0.11 to 0.00) | -0.07 (-0.13 to -0.01) |
| Stathmin-IF | 0.00 (-0.05 to 0.05) | 0.04 (0.00 to 0.07) | 0.04 (0.01 to 0.08) | 0.04 (0.00 to 0.07) | 0.05 (0.01 to 0.09) | 0.04 (0.00 to 0.07) | 0.05 (0.01 to 0.08) |
| pS6-IF | -0.06 (-0.11 to -0.01) | -0.05 (-0.09 to 0.00) | -0.05 (-0.10 to 0.00) | -0.05 (-0.09 to 0.00) | -0.05 (-0.10 to 0.00) | -0.04 (-0.09 to 0.00) | -0.05 (-0.09 to 0.00) |
| TUNEL | -0.01 (-0.07 to 0.06) | 0.01 (-0.05 to 0.07) | 0.01 (-0.05 to 0.07) | 0.01 (-0.04 to 0.07) | -0.01 (-0.25 to 0.23) | 0.01 (-0.05 to 0.06) | 0.01 (-0.05 to 0.07) |
| Vitamin D R | -0.12 (-0.17 to -0.06) | -0.13 (-0.17 to -0.08) | -0.13 (-0.18 to -0.08) | -0.13 (-0.18 to -0.08) | -0.12 (-0.17 to -0.07) | -0.12 (-0.17 to -0.07) | -0.12 (-0.17 to -0.08) |

## Table 6

Biomarker levels and lethal disease according to batch effect correction method. Hazard ratios (with 95% confidence intervals) per 1 standard deviation increase in the biomarker (linear) from unadjusted Cox regression models are shown.

```
coxformatted_linear <- function(marker, index) {
  res <- pos_coxbroom(data = comb, 
                      exposure = if_else(index == 1,
                                         true = marker,
                                         false = paste0(marker, "_adj", index))) %>%
    as.character()
  rowtitle <- model_labels[index]
  tibble(!!rowtitle := paste0(res[2], " (", res[3], "-", res[4], ")"))
}

markers %>%
  select(var_use, Marker = name) %>%
  mutate(res = map(.x = var_use, 
                   .f = ~map2_dfc(.x = .x, .y = 1:7, 
                                  .f = coxformatted_linear))) %>%
  unnest(cols = res) %>%
  select(-var_use) %>%
  mygt()
```

| Marker | 1 Uncorrected | 2 Simple mean | 3 Standardized mean | 4 IP-weighted mean | 5 Quantile regression | 6 Quantile normalization | 7 ComBat |
| --- | --- | --- | --- | --- | --- | --- | --- |
| Adiponectin R2 | 1.33 (1.12-1.59) | 1.49 (1.22-1.82) | 1.49 (1.22-1.82) | 1.49 (1.22-1.81) | 1.57 (1.26-1.95) | 1.57 (1.27-1.95) | 1.51 (1.24-1.85) |
| Androgen R | 1.02 (0.84-1.24) | 0.99 (0.79-1.25) | 0.98 (0.77-1.24) | 0.99 (0.78-1.24) | 0.97 (0.79-1.19) | 0.99 (0.77-1.27) | 0.98 (0.77-1.24) |
| Beta-catenin | 0.84 (0.70-1.01) | 0.82 (0.68-0.99) | 0.80 (0.67-0.97) | 0.82 (0.68-0.99) | 0.74 (0.58-0.94) | 0.82 (0.68-0.99) | 0.80 (0.66-0.97) |
| Calcium SR | 1.17 (1.00-1.37) | 0.97 (0.79-1.21) | 0.98 (0.79-1.21) | 0.97 (0.79-1.21) | 0.95 (0.79-1.14) | 1.00 (0.80-1.26) | 1.02 (0.82-1.26) |
| Cyclin D1 | 1.06 (0.87-1.31) | 1.15 (0.93-1.41) | 1.16 (0.94-1.43) | 1.16 (0.95-1.43) | 1.20 (0.97-1.49) | 1.12 (0.91-1.38) | 1.15 (0.93-1.41) |
| ER-alpha | 0.97 (0.80-1.18) | 0.78 (0.58-1.05) | 0.79 (0.59-1.05) | 0.78 (0.58-1.05) | 0.84 (0.68-1.05) | 0.83 (0.62-1.12) | 0.78 (0.58-1.04) |
| ER-beta | 1.21 (1.03-1.43) | 0.95 (0.75-1.20) | 0.97 (0.77-1.23) | 0.95 (0.75-1.20) | 0.99 (0.78-1.26) | 0.99 (0.78-1.26) | 0.98 (0.77-1.24) |
| FASN | 1.12 (0.92-1.36) | 1.15 (0.92-1.43) | 1.15 (0.92-1.44) | 1.14 (0.91-1.42) | 1.11 (0.90-1.38) | 1.13 (0.90-1.43) | 1.14 (0.91-1.42) |
| IGF1 R | 1.06 (0.85-1.31) | 0.97 (0.77-1.21) | 0.96 (0.76-1.19) | 0.94 (0.76-1.18) | 0.91 (0.74-1.12) | 0.95 (0.74-1.21) | 0.95 (0.76-1.19) |
| Insulin R | 0.94 (0.76-1.16) | 0.92 (0.74-1.14) | 0.92 (0.74-1.14) | 0.90 (0.73-1.13) | 0.89 (0.72-1.11) | 0.93 (0.74-1.16) | 0.91 (0.73-1.13) |
| Ki-67 | 1.51 (1.24-1.84) | 1.63 (1.33-2.01) | 1.58 (1.29-1.93) | 1.59 (1.30-1.94) | 1.50 (1.25-1.81) | 1.61 (1.32-1.97) | 1.56 (1.27-1.91) |
| MYC | 0.95 (0.76-1.20) | 0.92 (0.72-1.17) | 0.91 (0.71-1.17) | 0.92 (0.72-1.17) | 0.86 (0.63-1.16) | 0.88 (0.68-1.14) | 0.91 (0.71-1.16) |
| Osteopontin | 2.45 (1.84-3.26) | 2.08 (1.58-2.72) | 2.12 (1.61-2.79) | 2.04 (1.56-2.67) | 2.11 (1.56-2.86) | 2.22 (1.66-2.97) | 2.15 (1.62-2.85) |
| PSMA | 1.35 (1.12-1.63) | 1.39 (1.15-1.67) | 1.40 (1.16-1.68) | 1.39 (1.15-1.68) | 1.57 (1.22-2.00) | 1.42 (1.17-1.71) | 1.40 (1.16-1.69) |
| PTEN-IF | 0.65 (0.50-0.85) | 0.59 (0.45-0.77) | 0.57 (0.44-0.75) | 0.59 (0.45-0.77) | 0.55 (0.40-0.75) | 0.56 (0.42-0.75) | 0.57 (0.44-0.76) |
| SMAD4 | 1.02 (0.82-1.26) | 1.02 (0.82-1.27) | 1.00 (0.80-1.25) | 1.01 (0.81-1.26) | 0.96 (0.77-1.20) | 1.03 (0.82-1.29) | 1.02 (0.82-1.28) |
| Stathmin-IF | 1.44 (1.21-1.70) | 1.45 (1.13-1.86) | 1.40 (1.09-1.80) | 1.45 (1.13-1.86) | 1.41 (1.11-1.78) | 1.50 (1.15-1.96) | 1.50 (1.16-1.93) |
| pS6-IF | 1.02 (0.83-1.26) | 0.94 (0.76-1.16) | 0.95 (0.77-1.17) | 0.94 (0.76-1.16) | 0.93 (0.75-1.14) | 0.90 (0.72-1.13) | 0.93 (0.75-1.15) |
| TUNEL | 1.05 (0.84-1.31) | 1.03 (0.82-1.30) | 1.04 (0.83-1.31) | 1.04 (0.83-1.31) | 1.02 (0.96-1.08) | 1.06 (0.81-1.39) | 1.04 (0.82-1.31) |
| Vitamin D R | 0.75 (0.60-0.92) | 0.55 (0.42-0.71) | 0.54 (0.42-0.70) | 0.53 (0.41-0.69) | 0.53 (0.41-0.68) | 0.56 (0.43-0.72) | 0.54 (0.42-0.70) |

## Table 7

Biomarker levels and lethal disease according to batch effect correction method. Unlike in the preceding table, the hazard ratios (with 95% confidence intervals) are contrasts comparing extreme quartiles (fourth compared to first quartile) from unadjusted Cox regression models.

```
coxformatted_quartile <- function(marker, index) {
  exposure <- if_else(index == 1,
                      true = marker,
                      false = paste0(marker, "_adj", index))
  
  res <- coxbroom(data = comb %>%
                        mutate(quartiles = factor(as.numeric(
                          factor(Hmisc::cut2(x = get(exposure), 
                                             g = 4))))),
                      exposure = "quartiles") %>%
    filter(term == "quartiles4") %>%
    as.character()
  rowtitle <- model_labels[index]
  tibble(!!rowtitle := paste0(res[2], " (", res[3], "-", res[4], ")"))
}

markers %>%
  select(var_use, Marker = name) %>%
  mutate(res = map(.x = var_use, 
                   .f = ~map2_dfc(.x = .x, .y = 1:7, 
                                  .f = coxformatted_quartile))) %>%
  unnest(cols = res) %>%
  select(-var_use) %>%
  mygt()
```

| Marker | 1 Uncorrected | 2 Simple mean | 3 Standardized mean | 4 IP-weighted mean | 5 Quantile regression | 6 Quantile normalization | 7 ComBat |
| --- | --- | --- | --- | --- | --- | --- | --- |
| Adiponectin R2 | 1.91 (1.12-3.27) | 2.93 (1.64-5.24) | 2.93 (1.64-5.24) | 3.20 (1.79-5.72) | 2.38 (1.35-4.19) | 2.98 (1.69-5.27) | 2.43 (1.37-4.31) |
| Androgen R | 1.10 (0.61-1.97) | 1.01 (0.58-1.73) | 0.97 (0.56-1.68) | 1.06 (0.61-1.84) | 0.98 (0.57-1.70) | 0.94 (0.55-1.60) | 0.96 (0.56-1.67) |
| Beta-catenin | 0.54 (0.30-0.95) | 0.50 (0.27-0.91) | 0.42 (0.23-0.79) | 0.50 (0.27-0.91) | 0.36 (0.18-0.71) | 0.45 (0.23-0.86) | 0.45 (0.24-0.85) |
| Calcium SR | 1.45 (0.92-2.27) | 1.21 (0.76-1.93) | 1.21 (0.76-1.93) | 1.21 (0.76-1.93) | 0.98 (0.63-1.52) | 1.04 (0.65-1.66) | 1.35 (0.83-2.17) |
| Cyclin D1 | 1.00 (0.58-1.72) | 1.08 (0.63-1.86) | 1.08 (0.62-1.86) | 1.17 (0.68-2.03) | 1.11 (0.65-1.90) | 1.11 (0.65-1.90) | 1.13 (0.65-1.95) |
| ER-alpha | 0.81 (0.47-1.40) | 0.66 (0.37-1.18) | 0.70 (0.40-1.20) | 0.66 (0.37-1.18) | 0.70 (0.41-1.18) | 0.65 (0.38-1.11) | 0.62 (0.35-1.09) |
| ER-beta | 1.46 (0.86-2.47) | 0.82 (0.48-1.39) | 0.94 (0.56-1.58) | 0.82 (0.48-1.39) | 1.01 (0.59-1.73) | 0.96 (0.56-1.63) | 1.05 (0.62-1.78) |
| FASN | 1.27 (0.72-2.24) | 1.13 (0.65-1.98) | 1.18 (0.68-2.04) | 1.04 (0.59-1.84) | 1.03 (0.60-1.78) | 1.11 (0.65-1.92) | 1.12 (0.65-1.91) |
| IGF1 R | 1.11 (0.64-1.94) | 0.83 (0.46-1.47) | 0.83 (0.46-1.47) | 0.82 (0.46-1.46) | 0.92 (0.52-1.62) | 0.85 (0.48-1.51) | 0.83 (0.47-1.48) |
| Insulin R | 0.86 (0.39-1.91) | 0.87 (0.45-1.67) | 0.87 (0.45-1.67) | 0.86 (0.45-1.66) | 0.75 (0.39-1.43) | 0.76 (0.40-1.43) | 0.76 (0.40-1.44) |
| Ki-67 | 2.44 (1.47-4.07) | 3.23 (1.85-5.64) | 2.93 (1.67-5.15) | 2.42 (1.48-3.95) | 2.42 (1.43-4.09) | 2.92 (1.69-5.05) | 2.11 (1.28-3.48) |
| MYC | 0.93 (0.50-1.71) | 1.03 (0.54-1.97) | 0.97 (0.51-1.84) | 1.03 (0.54-1.97) | 0.82 (0.43-1.53) | 0.83 (0.44-1.57) | 0.93 (0.49-1.77) |
| Osteopontin | 5.55 (2.80-10.98) | 5.07 (2.46-10.45) | 4.70 (2.35-9.39) | 4.18 (2.08-8.40) | 4.32 (2.22-8.41) | 4.62 (2.39-8.95) | 4.79 (2.40-9.56) |
| PSMA | 2.20 (1.25-3.86) | 2.65 (1.45-4.83) | 2.24 (1.26-3.99) | 2.63 (1.45-4.80) | 2.34 (1.32-4.15) | 2.44 (1.38-4.33) | 2.49 (1.39-4.49) |
| PTEN-IF | 0.41 (0.22-0.74) | 0.36 (0.19-0.66) | 0.37 (0.20-0.69) | 0.36 (0.19-0.66) | 0.31 (0.16-0.60) | 0.33 (0.18-0.62) | 0.32 (0.17-0.60) |
| SMAD4 | 1.05 (0.59-1.84) | 0.80 (0.46-1.40) | 0.69 (0.39-1.23) | 0.91 (0.51-1.61) | 0.95 (0.52-1.71) | 0.91 (0.51-1.64) | 0.75 (0.43-1.31) |
| Stathmin-IF | 1.85 (1.04-3.28) | 2.11 (1.20-3.72) | 1.75 (1.00-3.05) | 2.11 (1.20-3.72) | 1.63 (0.91-2.90) | 1.80 (1.02-3.18) | 1.64 (0.94-2.84) |
| pS6-IF | 1.25 (0.71-2.18) | 0.86 (0.48-1.56) | 0.91 (0.51-1.62) | 0.86 (0.48-1.56) | 0.84 (0.48-1.47) | 0.84 (0.48-1.47) | 1.01 (0.57-1.79) |
| TUNEL | 1.11 (0.59-2.07) | 1.13 (0.61-2.10) | 1.17 (0.64-2.16) | 1.16 (0.63-2.14) | 0.94 (0.49-1.81) | 1.14 (0.62-2.13) | 1.53 (0.81-2.88) |
| Vitamin D R | 0.44 (0.23-0.86) | 0.19 (0.09-0.41) | 0.19 (0.09-0.42) | 0.19 (0.09-0.40) | 0.17 (0.08-0.37) | 0.19 (0.09-0.40) | 0.20 (0.09-0.43) |

# Data in Text

## Basic counts

Summaries of patients/tumors per TMA

```
comb %>%
  group_by(tma) %>%
  summarize(`n of tumors` = n(),
            `Median dx year` = median(dxyear),
            `% Gleason 8+` = sum(if_else(gleason %in% c("8", "9-10"), 1, 0)) / n() * 100,
            `Lethal rate / 1000 py` = sum(eventlethal) / sum(lethalfu) * 12 * 1000,
            .groups = "drop") %>%
  select(-tma) %>%
  pivot_longer(cols = everything(), names_to = "Characteristic") %>%
  group_by(Characteristic) %>%
  summarize_all(.funs = list(Min = min, Max = max, Range = ~max(.)-min(.))) %>%
  mutate_if(.predicate = is.numeric,
            .funs = round) %>%
  mygt()
```

| Characteristic | Min | Max | Range |
| --- | --- | --- | --- |
| % Gleason 8+ | 11 | 33 | 21 |
| Lethal rate / 1000 py | 2 | 16 | 14 |
| Median dx year | 1992 | 2006 | 14 |
| n of tumors | 47 | 158 | 111 |

Tumors/participants with at least one biomarker

```
comb %>% nrow()
```

```
## [1] 1448
```

Tumors/participants with at least one biomarker, restricted to tumors with Gleason score 3+4, stage pT1/T2, and tissue source prostatectomy (because pTNM is not missing)–the largest three-way combination these variables

```
comb %>% 
  filter(gleason == "3+4" & 
           ptnm_miss2 == "pT1/T2") %>% 
  nrow()
```

```
## [1] 378
```

## ICCs

Between-TMA differences for ER-alpha

```
comb %>%
  group_by(tma) %>%
  summarize(mean = mean(er_alpha_intens, na.rm = TRUE),
            var  = var(er_alpha_intens, na.rm = TRUE),
            .groups = "drop") %>%
  select(-tma) %>%
  summarize_all(.funs = list(min = min, max = max)) %>%
  mutate(mean_diff  = mean_max - mean_min,
         var_ratio  = var_max / var_min) %>%
  mutate_all(.funs = ~format(round(., digits = 2), nsmall = 2)) %>%
  mygt()
```

| mean\_min | var\_min | mean\_max | var\_max | mean\_diff | var\_ratio |
| --- | --- | --- | --- | --- | --- |
| -0.96 | 0.14 | 1.25 | 1.35 | 2.21 | 9.34 |

ICCs across all markers

```
markersfig2 %>% tsummary(ICC) %>% 
  select(-rows, -distin, -sd, -sum) %>% 
  mutate_at(.vars = vars(-variable, -obs),
            .funs = ~format(round(., digits = 2), nsmall = 2)) %>%
  mygt()
```

| variable | obs | min | q25 | median | q75 | max | mean |
| --- | --- | --- | --- | --- | --- | --- | --- |
| ICC | 20 | 0.01 | 0.06 | 0.12 | 0.26 | 0.48 | 0.17 |

ICCs after restriction to Gleason score 3+4, pT1/T2

```
icc_restrict %>% 
  tsummary(ICC) %>% 
  select(-rows, -distin, -sd, -sum) %>% 
  mutate_at(.vars = vars(-variable, -obs),
            .funs = ~format(round(., digits = 2), nsmall = 2)) %>%
  mygt()
```

| variable | obs | min | q25 | median | q75 | max | mean |
| --- | --- | --- | --- | --- | --- | --- | --- |
| ICC | 20 | 0.00 | 0.05 | 0.18 | 0.29 | 0.50 | 0.19 |

ICCs for Gleason grade

```
icc_gleason %>% 
  tsummary(ICC) %>% 
  select(-rows, -distin, -sd, -sum) %>% 
  mutate_at(.vars = vars(-variable, -obs),
            .funs = ~format(round(., digits = 2), nsmall = 2)) %>%
  mygt()
```

| variable | obs | min | q25 | median | q75 | max | mean |
| --- | --- | --- | --- | --- | --- | --- | --- |
| ICC | 20 | 0.00 | 0.00 | 0.01 | 0.02 | 0.13 | 0.02 |

```
icc_gleason %>%
  filter(var_use == "mean_psma_region_score") %>%
  select(-var_use) %>%
  mutate_at(.vars = vars(ICC, ll, ul),
            .funs = ~format(round(., digits = 2), nsmall = 2)) %>%
  mygt()
```

| name | ICC | ll | ul |
| --- | --- | --- | --- |
| PSMA | 0.13 | 0.01 | 0.28 |

## Principal components

% variance explained by first two principal components

```
comb %>% 
  select(markers$var_use) %>% 
  FactoMineR::PCA(graph = FALSE) %>%
  pluck("eig") %>%
  as_tibble(rownames = "principal_component") %>%
  slice(1:2) %>%
  mutate_if(.predicate = is.numeric, .funs = round) %>%
  mygt()
```

| principal\_component | eigenvalue | percentage of variance | cumulative percentage of variance |
| --- | --- | --- | --- |
| comp 1 | 4 | 18 | 18 |
| comp 2 | 2 | 9 | 27 |

## Core-level data

```
core_er %>% 
  group_by(marker) %>% 
  summarize(`Count of cores` = n(), 
            `Count of tumors` = length(unique(id)),
            .groups = "drop") %>%
  mygt()
```

| marker | Count of cores | Count of tumors |
| --- | --- | --- |
| ERalpha | 55 | 10 |
| ERbeta | 53 | 10 |

**Between-TMA ICCs accounting for between-core variance**

\(Y\_{ijk} = \beta\_0 + s\_i + r\_j + e\_{ijk}\)

where \(\beta\_0\), grand mean; \(s\_i\), tumor-specific random effect; \(r\_j\), TMA-specific random effect; \(e\_{ijk}\), residual error (per core).

\(ICC = \frac{var(r)}{var(r) + var(s) + var(e)}\)

This estimate corresponds to the “two-way random effects model” in the ICC literature.

“Manual” ICC calculation

```
core_er %>%
  filter(nuc_mean_z < 7) %>%  # exclude one very extreme ER-alpha core with z score of 8
  mutate(id = factor(id), tma = factor(tma)) %>%
  group_by(marker) %>%
  nest() %>%
  summarize(res = map(
    .x = data, 
    .f = ~{
      summary(lme4::lmer(formula = nuc_mean_z ~ 1 + (1 | id) + (1 | tma), 
                   data = .x)) %>% 
        pluck("varcor") %>% as_tibble() %>%
        mutate(icc = format(round(vcov / sum(vcov), digits = 2), nsmall = 2))
    }),
    .groups = "drop") %>%
  unnest(cols = res) %>%
  select(-var1, -var2) %>%
  mygt()
```

| marker | grp | vcov | sdcor | icc |
| --- | --- | --- | --- | --- |
| ERalpha | id | 0.6749535 | 0.8215555 | 0.37 |
| ERalpha | tma | 0.5432881 | 0.7370808 | 0.30 |
| ERalpha | Residual | 0.5927471 | 0.7699007 | 0.33 |
| ERbeta | id | 0.2534206 | 0.5034090 | 0.26 |
| ERbeta | tma | 0.2386415 | 0.4885095 | 0.24 |
| ERbeta | Residual | 0.4851763 | 0.6965460 | 0.50 |

Using `rptR::rpt()`, which also uses `lmer()` for mixed models. Includes bootstrapped 95% CIs

```
# TWO-way ICCs based on lmer() with bootstrapped CIs
icc_twoway <- function(data, marker) {
  set.seed(123)
  res <- rptR::rpt(formula  = as.formula(paste(marker, "~ (1 | tma) + (1 | id)")),
                   grname   = c("tma", "id"), 
                   data     = data, 
                   datatype = "Gaussian", 
                   nboot    = bootrepeat,  # in global environment
                   npermut  = 0, 
                   parallel = FALSE)  # disabled to allow script submission through shell
  bind_cols(res$CI_emp %>% as_tibble(rownames = "term"), 
            ICC = res$R %>% t() %>% as.numeric()) %>%
    select(term, ICC, ll = `2.5%`, ul = `97.5%`)
}

rpt_core_er <- core_er %>%
  filter(nuc_mean_z < 7) %>%  # exclude one very extreme ER-alpha core with z score of 8
  mutate(id = factor(id), tma = factor(tma)) %>%
  group_by(marker) %>%
  nest() %>%
  summarize(res = map(.x = data, .f = icc_twoway, marker = "nuc_mean_z"),
            .groups = "drop") %>%
  unnest(cols = res) %>%
  mutate_at(.vars = vars(ICC, ll, ul), .funs = ~format(round(., digits = 2), nsmall = 2))
```

```
rpt_core_er %>% mygt()
```

| marker | term | ICC | ll | ul |
| --- | --- | --- | --- | --- |
| ERalpha | tma | 0.30 | 0.00 | 0.67 |
| ERalpha | id | 0.37 | 0.06 | 0.68 |
| ERbeta | tma | 0.24 | 0.00 | 0.60 |
| ERbeta | id | 0.26 | 0.00 | 0.57 |

## ICC after correction

```
icc_after_summary <- markers %>%
  select(var_use) %>%
  expand_grid(tibble(method = 1:7,
                     adj = c("", paste0("_adj", 2:7)))) %>%
  mutate(exposure = paste0(var_use, adj),
         method = model_labels[method]) %>%
  filter(exposure %in% names(comb)) %>%
  mutate(icc = map_dbl(.x = exposure,
                       .f = ~rptR::rpt(formula  = as.formula(paste(.x, "~ (1 | tma)")),
                                       grname   = "tma", 
                                       data     = comb, 
                                       datatype = "Gaussian", 
                                       nboot    = 0,
                                       npermut  = 0, 
                                       parallel = FALSE)$R[1,1])) %>%
  group_by(method) %>%
  summarize_at(.vars = vars(icc),
               .funs = list(min = min, median = median, max = max)) %>%
  ungroup() %>%
  mutate_if(.predicate = is.numeric, 
            .funs = ~format(round(., digits = 2), nsmall = 2))
```

```
icc_after_summary %>% mygt()
```

| method | min | median | max |
| --- | --- | --- | --- |
| 1 Uncorrected | 0.01 | 0.12 | 0.48 |
| 2 Simple mean | 0.00 | 0.00 | 0.00 |
| 3 Standardized mean | 0.00 | 0.00 | 0.01 |
| 4 IP-weighted mean | 0.00 | 0.00 | 0.02 |
| 5 Quantile regression | 0.00 | 0.00 | 0.10 |
| 6 Quantile normalization | 0.00 | 0.00 | 0.00 |
| 7 ComBat | 0.00 | 0.00 | 0.01 |

```
sessionInfo()
```

```
## R version 4.0.5 (2021-03-31)
## Platform: x86_64-pc-linux-gnu (64-bit)
## Running under: CentOS Linux 7 (Core)
## 
## Matrix products: default
## BLAS:   /usr/lib64/libblas.so.3.4.2
## LAPACK: /usr/lib64/liblapack.so.3.4.2
## 
## locale:
##  [1] LC_CTYPE=en_US.UTF-8       LC_NUMERIC=C              
##  [3] LC_TIME=en_US.UTF-8        LC_COLLATE=en_US.UTF-8    
##  [5] LC_MONETARY=en_US.UTF-8    LC_MESSAGES=en_US.UTF-8   
##  [7] LC_PAPER=en_US.UTF-8       LC_NAME=C                 
##  [9] LC_ADDRESS=C               LC_TELEPHONE=C            
## [11] LC_MEASUREMENT=en_US.UTF-8 LC_IDENTIFICATION=C       
## 
## attached base packages:
## [1] stats     graphics  grDevices utils     datasets  methods   base     
## 
## other attached packages:
##  [1] risks_0.2.2-9005  khsmisc_0.4.0     gt_0.3.1          viridis_0.5.1    
##  [5] viridisLite_0.3.0 cowplot_1.1.1     survival_3.2-10   readxl_1.3.1     
##  [9] labelled_2.7.0    broom_0.7.9       ggplot2_3.3.3     stringr_1.4.0    
## [13] purrr_0.3.4       readr_1.4.0       forcats_0.5.1     tibble_3.1.0     
## [17] tidyr_1.1.3       dplyr_1.0.4       khsverse_0.0.2    batchtma_0.1.5   
## [21] here_1.0.1       
## 
## loaded via a namespace (and not attached):
##   [1] utf8_1.2.1           tidyselect_1.1.0     lme4_1.1-26         
##   [4] RSQLite_2.2.5        AnnotationDbi_1.50.3 htmlwidgets_1.5.3   
##   [7] FactoMineR_2.4       grid_4.0.5           combinat_0.0-8      
##  [10] BiocParallel_1.22.0  munsell_0.5.0        codetools_0.2-18    
##  [13] statmod_1.4.35       DT_0.17              withr_2.4.1         
##  [16] colorspace_2.0-0     Biobase_2.48.0       highr_0.8           
##  [19] knitr_1.31           rstudioapi_0.13      leaps_3.1           
##  [22] stats4_4.0.5         ggsignif_0.6.1       labeling_0.4.2      
##  [25] KMsurv_0.1-5         bit64_4.0.5          farver_2.1.0        
##  [28] rprojroot_2.0.2      coda_0.19-4          vctrs_0.3.6         
##  [31] generics_0.1.0       xfun_0.21            geepack_1.3-2       
##  [34] R6_2.5.0             doParallel_1.0.16    locfit_1.5-9.4      
##  [37] bitops_1.0-6         cachem_1.0.4         addreg_3.0          
##  [40] assertthat_0.2.1     scales_1.1.1         nnet_7.3-15         
##  [43] gtable_0.3.0         sva_3.36.0           conquer_1.0.2       
##  [46] rptR_0.9.22          rlang_0.4.10         MatrixModels_0.5-0  
##  [49] genefilter_1.70.0    scatterplot3d_0.3-41 splines_4.0.5       
##  [52] rstatix_0.7.0        TMB_1.7.19           checkmate_2.0.0     
##  [55] yaml_2.2.1           reshape2_1.4.4       abind_1.4-5         
##  [58] modelr_0.1.8         turboEM_2020.1       gtsummary_1.4.2     
##  [61] backports_1.2.1      Hmisc_4.5-0          tools_4.0.5         
##  [64] ellipsis_0.3.1       RColorBrewer_1.1-2   jquerylib_0.1.3     
##  [67] BiocGenerics_0.36.1  Rcpp_1.0.7           plyr_1.8.6          
##  [70] base64enc_0.1-3      RCurl_1.98-1.3       rpart_4.1-15        
##  [73] ggpubr_0.4.0         pbapply_1.4-3        S4Vectors_0.26.1    
##  [76] zoo_1.8-9            haven_2.3.1          ggrepel_0.9.1       
##  [79] cluster_2.1.1        fs_1.5.0             magrittr_2.0.1      
##  [82] data.table_1.14.0    openxlsx_4.2.3       SparseM_1.81        
##  [85] reprex_1.0.0         survminer_0.4.8      matrixStats_0.58.0  
##  [88] hms_1.0.0            evaluate_0.14        xtable_1.8-4        
##  [91] XML_3.99-0.6         jpeg_0.1-8.1         rio_0.5.26          
##  [94] broom.mixed_0.2.6    IRanges_2.22.2       gridExtra_2.3       
##  [97] compiler_4.0.5       crayon_1.4.1         minqa_1.2.4         
## [100] htmltools_0.5.1.1    mgcv_1.8-34          Formula_1.2-4       
## [103] lubridate_1.7.10     DBI_1.1.1            dbplyr_2.1.0        
## [106] MASS_7.3-53.1        broom.helpers_1.3.0  boot_1.3-27         
## [109] Matrix_1.3-2         car_3.0-10           cli_2.5.0           
## [112] parallel_4.0.5       pkgconfig_2.0.3      km.ci_0.5-2         
## [115] logbin_2.0.4         flashClust_1.01-2    numDeriv_2016.8-1.1 
## [118] foreign_0.8-81       xml2_1.3.2           foreach_1.5.1       
## [121] annotate_1.66.0      bslib_0.2.4          rvest_0.3.6         
## [124] digest_0.6.27        rmarkdown_2.7        cellranger_1.1.0    
## [127] htmlTable_2.1.0      survMisc_0.5.5       glm2_1.2.1          
## [130] edgeR_3.30.3         curl_4.3             quantreg_5.85       
## [133] nloptr_1.2.2.2       lifecycle_1.0.0      nlme_3.1-152        
## [136] jsonlite_1.7.2       carData_3.0-4        limma_3.44.3        
## [139] fansi_0.4.2          pillar_1.5.0         lattice_0.20-41     
## [142] fastmap_1.1.0        httr_1.4.2           glue_1.4.2          
## [145] zip_2.1.1            png_0.1-7            iterators_1.0.13    
## [148] bit_4.0.4            stringi_1.5.3        sass_0.3.1          
## [151] blob_1.2.1           latticeExtra_0.6-29  memoise_2.0.0       
## [154] tidyverse_1.3.0
```
